# Evolution and Function of the Notch Signaling Pathway: An Invertebrate Perspective

**DOI:** 10.3390/ijms25063322

**Published:** 2024-03-15

**Authors:** Yan Lv, Xuan Pang, Zhonghong Cao, Changping Song, Baohua Liu, Weiwei Wu, Qiuxiang Pang

**Affiliations:** 1 Anti-Aging & Regenerative Medicine Research Institution, School of Life Sciences and Medicine, Shandong University of Technology, Zibo 255049, China; 19956846993@163.com (Y.L.); pangxuan0307@163.com (X.P.); zhcao@sdut.edu.cn (Z.C.); ppliew@szu.edu.cn (B.L.); 2 Jiangsu Province Hi-Tech Key Laboratory for Biomedical Research, School of Chemistry and Chemical Engineering, Southeast University, Nanjing 211189, China; songchangping@163.com; 3 Shenzhen Key Laboratory for Systemic Aging and Intervention (SKL-SAI), School of Basic Medical Sciences, Shenzhen University, Shenzhen 518060, China

**Keywords:** invertebrates, Notch signaling pathway, γ-secretase, neurogenesis, regeneration

## Abstract

The highly conserved Notch signaling pathway affects embryonic development, neurogenesis, homeostasis, tissue repair, immunity, and numerous other essential processes. Although previous studies have demonstrated the location and function of the core components of Notch signaling in various animal phyla, a more comprehensive summary of the Notch core components in lower organisms is still required. In this review, we objectively summarize the molecular features of the Notch signaling pathway constituents, their current expression profiles, and their functions in invertebrates, with emphasis on their effects on neurogenesis and regeneration. We also analyze the evolution and other facets of Notch signaling and hope that the contents of this review will be useful to interested researchers.

## 1. Introduction

The Notch gene was discovered over a century ago and was named after the Notched wing phenotype of *Drosophila melanogaster* (*D. melanogaster*) [1,2]. It was later extensively studied in vertebrates and invertebrates. The Notch gene encodes a transmembrane receptor that gives its name to the evolutionarily highly conserved Notch signaling cascade, a well-preserved pathway that regulates cell fate through cell–cell communication. It plays a pivotal role in regulating multiple fundamental cellular processes, such as proliferation, stem cell maintenance, and differentiation throughout embryonic and adult development. Consequently, under- or mis-regulation of Notch signaling is at the root of a wide range of human disorders, such as developmental syndromes and adult-onset cancers.

Activation of the Notch receptor is irreversible because it involves a proteolysis-mediated release of the Notch intracellular domain (NICD), which translocates to the nucleus and associates with a DNA-bound protein. Three primary modes of Notch actions have been discovered: lateral inhibition, boundary induction, and lineage decision [3]. Although each Notch molecule only signals once without being amplified by secondary messengers, Notch signaling is surprisingly robust in most tissues [4]. It is known that the loss of Notch signaling leads to a series of mutant phenotypes in metazoans [5]. However, only limited information has been available on the regulatory mechanisms of the Notch signaling pathway in non-model invertebrates. Here, we give a brief introduction to the Notch signaling pathway, summarize the expression profiles of the relevant Notch signaling molecules, and show how Notch signaling members orchestrate neurodevelopment, tissue regeneration, and embryonic development in several selected invertebrates, especially in aquatic organisms.

## 2. The Architecture of the Canonical Notch Signaling Pathway

**Figure 1:**
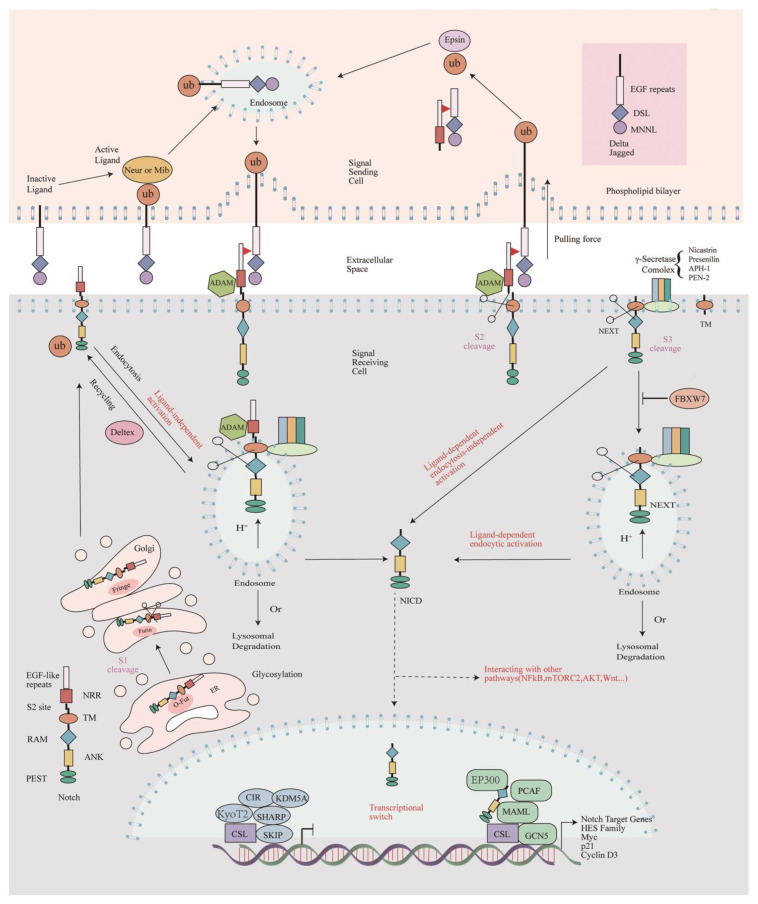
Major components and auxiliary factors of the Notch signaling pathway (modified from [6]). In signal-receiving cells, Notch receptors are first synthesized in the endoplasmic reticulum and then translocated to the Golgi apparatus, where EGF-like repeat sequences undergo glycosylation modification during translocation. The Notch receptor then undergoes S1 cleavage in the Golgi to form a heterodimer and is transported to the cell membrane. In the presence of ubiquitin ligase, some Notch receptors on the cell membrane are endocytosed into the nuclear endosome, which has an acidic environment and contains ADAMs and γ-secretase. Notch receptors in endosomes have two fates, they can either be recirculated to the cell membrane and cleaved to the NICD (the E3 ubiquitin ligase Deltex acts as an activator of Notch, capable of upregulating Notch), or they are translocated to lysosomes for degradation. In signaling cells, the Notch ligand is inactive at the cell membrane, and after Neur or Mib ubiquitination, the ligand is activated and a Notch signaling cascade occurs (ligand–receptor binding indicated by bright red triangles). The ligand can then be endocytosed and the accompanying pulling force induces traction of the bound ligand and receptor toward the side of the signaling cell. In the absence of traction, the S2 site of the Notch receptor is masked by the NRR, and with increasing traction, the S2 cleavage site is exposed, allowing for a second cleavage to occur. ADAMs are indispensable for S2 cleavage. The product of Notch undergoing S2 cleavage is NEXT, which also has two fates, either remaining on the cell membrane and binding to γ-secretase to undergo a third cleavage (S3 cleavage) to produce the NICD, or endocytosis into the endosome and binding to γ-secretase on the membrane to undergo cleavage to produce either NICD or NEXT translocation to the lysosome for degradation. In total, there are three approaches to generate the NICD, which are classified as ligand-independent activation, ligand-dependent endocytosis-independent activation, and ligand-dependent endocytic activation. The resulting NICD can undergo phosphorylation modifications, leading to FBXW7-mediated NICD degradation. The NICD can translocate into the nucleus or remain in the cytoplasm to crosstalk with other signaling pathways such as NFκB, mTORC2, AKT, and Wnt. In the absence of the NICD, CSL binds to transcriptional repressors to inhibit the transcription of target genes. Once the NICD enters the nucleus, it can bind to CSL to recruit MAML, release transcriptional repressors, and recruit coactivators to promote the transcription of Notch target genes. The top right corner is the structural view of the ligand and the bottom left corner is the structural view of the receptor. NICD, Notch intracellular domain; ADAM, a disintegrin and metalloproteinase domain-containing protein; Neur, Neuralized; Mib, Mindbomb; NRR, negative regulatory region; NEXT, Notch extracellular truncation; CSL, Rbpj/Su(H)/Lag1; MAML, Mastermind-like proteins; TM, transmembrane domain; RAM, RBPJ association module; ANK, ankyrin repeats; PEST, proline/glutamic acid/serine/threonine-rich motifs; NLS, nuclear localization sequence; KyoT2/CIR/SHARP/SKIP/KDM5A, transcriptional repressor; EP300/MAML/PCAF/GCN5, transcription activator.

The Notch pathway’s molecular architecture is straightforward (Figure 1 modified from [6]). Signaling within the pathway takes place when the ligands present on the surface of a cell interact with the Notch receptor situated on a neighboring cell. Upon binding with specific ligands, the intracellular portion of the Notch receptor (NICD or Notch ICDs) is cleaved by γ-secretase. It is then transported to the nucleus, where it binds with the transcription factor recombining binding protein J-kappa (RBP-J) to regulate gene transcription. The same ligands can also interact with the Notch receptor within the same cell (in cis), but this cis-interaction causes a cis-inhibition of Notch, which reduces a cell’s ability to receive the signal from neighboring cells [7].

### 2.1. Notch Receptor Processing and Signal Transduction

Notch proteins transmit signals through three-step proteolytic cleavage [8]. After the synthesis of the Notch protein, it will enter the endoplasmic reticulum (ER) and the Golgi apparatus for glycosylation modification, which is vital to the stability and function of Notch. Subsequently, the S1 site of the Notch protein is cleaved (S1 cleavage) by a furin-like proteolytic enzyme in the Golgi body, converting Notch into a mature heterodimeric form. After this first cleavage, the Notch protein will enter the surface of the cell membrane in the form of a heterodimer [9]. Classically, the mature Notch protein is composed of an extracellular domain (NECD) containing multiple EGF and LNR motifs, NICD-containing ankyrin repeat (ANK) domains, and a PEST region [10].

The number of Notch receptor genes, Notch ligand genes, and Hes family genes is extremely variable, indicating that the evolution of these genes in each animal clade has been complex, with numerous independent gene duplications, or numerous gene losses, or a combination of both phenomena. For example, a single Notch gene is found in most species, with the exception of vertebrates (2 to 4 genes), the annelid *Helobdella* (2 genes), and the planarian (6 genes) [10]. In the common animals *Drosophila melanogaster* (*D. melanogaster*), *Caenorhabditis elegans* (*C. elegans*), and mammals, there are one (Notch), two (Lin-12 and Glp-1), and four (Notch1-4) Notch paralogs, respectively. Meanwhile, in *D. melanogaster* and mammals, two Notch ligands [Delta (Dl) and Serrate (Ser)] and five acknowledged Notch ligands (e.g., Jagged 1, Jagged 2, Delta-like ligand 1, Delta-like ligand 3, Delta-like ligand 4) have been identified, respectively. All receptors and ligands have redundant and unique functions in many different aspects of metazoan life. In the canonical Notch pathway, binding the extracellular domains between ligands and Notch receptors is vital for signal initiation. Binding between Notch and its ligands is based on its extracellular domain structures, which contain tandem epidermal growth factor (EGF)-like repeats that are responsible for ligand binding. It is hypothesized that Notch family members may have originally evolved as cell adhesion molecules to mediate multicellularity in the last common ancestor of metazoans. And studies in the fly *D. melanogaster* and in mammals have shown that Notch mediates cell adhesion and that this function may precede its signaling function. However, further studies are needed to confirm this suggestion [11].

When the ligands bind, a mechanical pulling force is thought to expose a second cleavage site (S2 site) for S2 cleavage in the Notch negative regulatory region (NRR) domain due to conformational changes that occur within the Lin-12/Notch repeat region (LNR) domain [12]. This S2 site is catalyzed by members of the ADAM (a disintegrin and metalloprotease) family of metalloproteases [13] and give rise to the Notch extracellular truncation (NEXT), which contains only the transmembrane and the intracellular domains. Subsequently, the NICD of the NEXT is cleaved at the S3 site by the γ-secretase complex (see Section 2.2 of this review), a process known as S3 cleavage, which occurs both at the cell membrane and in the endosome after NEXT is endocytosed.

Once cleaved off from the membrane, the NICD is translocated into the nucleus without any intermediates, where it binds directly to the transcription factor RBPJ, a protein also known as CSL/CBF1 in mammals, Su(H) in flies, and Lag-1 in worms. In the absence of the NICD, RBPJ binds a consensus site (GTGGGA) within the promoters of the Notch target genes and inhibits gene expression by recruiting transcriptional repressors [6]. In the presence of the NICD, however, it forms a trimeric complex with RBPJ and Mastermind-like protein (MAML). This trimeric complex recruits several additional coactivators (see Section 2.3.1 in this review) that lead to transcriptional activation of the Notch target genes. Classical Notch target genes include Hairy/Enhancer of Split [Hes/E(Spl)] and Hes-related family BHLH transcription factor with YRPW motif (Hey), two gene families that encode repressive basic helix–ring–helix (bHLH) proteins [14]. New models suggest that NICD does not orchestrate a synchronous transcriptional response in the nucleus. Instead, NICD promotes the opening state of chromatin, causing the NICD-containing RBPJ complex to bind only to loose chromatin, whereas the NICD-free RBPJ complex binds both compact and loose chromatin [15]. The presence of more NICD-containing RBPJ complexes promotes gene expression. However, details of NICD translocation and transcriptional regulation remain to be elucidated.

### 2.2. The Notch Cascade Is Negatively Affected by γ-Secretase Inhibitor

From the brief introduction to the Notch pathway mentioned above, we know that the enzymes that catalyze Notch glycosylation and cleavage, the genes that interact with RBPJ to regulate transcription, are all potential targets for Notch cascade inhibitors. Among these, the γ-secretase inhibitor N-[N-(3,5-difluorophenacetyl)-L-alanyl]-S-phenylglycine t-butyl ester (DAPT) is widely used and it inhibits Notch activation by preventing the γ-secretase-mediated cleavage of Notch and release of the NICD [16].

γ-secretase is an intramembrane proteolytic complex containing four essential subunits: the catalytic subunit presenilin (Ps), the mono-transmembrane protein nicastrin (Nct), anterior pharynx-defective 1 (Aph-1), and presenilin enhancer 2 (Pen-2) [17]. In order to exert their normal physiological functions, the four proteins must be properly assembled, matured through secretory pathways, and transported to the appropriate site. Changes in the expression levels of any one of these proteins will affect the stability, hydrolysis, maturation, or transport function of other proteins to the cell surface, thereby affecting the activity of the γ-secretase.

γ-secretase cleaves the transmembrane helices of type I membrane protein substrates [18]. More than 90 transmembrane proteins have been reported as substrates for γ-secretase. Two of the best understood and studied of these substrates are amyloid precursor protein (APP) and Notch receptor, which are precursors for the formation of amyloid beta (Aβ) and the NICD, respectively [19]. Therefore, understanding the mechanism of substrate recognition by γ-secretase is helpful in the development of inhibitors, while the discovery of substrate-specific inhibitors of Notch is underway, benefiting from the research on the structural information. And effective γ-secretase inhibitors (GSIs) are of great help in the treatment of clinical diseases.

### 2.3. The Auxiliary Proteins That Affect Notch Signaling

The output of canonical Notch signaling is modulated at several levels. In addition to the relative levels of Notch protein, ligands, or different Notch components, other post-translational modifications of the receptors and ligands also tune the amplitude and timing of Notch activity to generate context-specific signals. Meanwhile, factors that influence the strength of the Notch cascade also include many tissue-specific transcriptional regulator molecules. These accessory proteins involved in the Notch signaling pathway can be divided into two groups: those that inhibit signaling and those that promote signaling.

#### 2.3.1. Corepressors and Coactivators of Rbpj

Diverse components directly interact with RBPJ proteins to form nuclear inhibitory complexes within specific cells, tissues, and different developmental states to negatively regulate Notch-dependent gene transcription. These several different corepressors include Ski-interacting protein (SKIP), CBF1-interacting corepressor (CIR), KyoT2, *Drosophila* Hairless, Split Ends [SPEN, also known as Msx2-interacting nuclear target protein (MINT) or SMRT/HDAC1-associated repressor protein (SHARP)], as well as the histone demethylase lysine (k)-specific demethylase 5A (KDM5A), and so on [20,21].

Whereas in the presence of the NICD, the CSL-NICD-MAML transactivation complex recruits histone-modifying coactivators such as CREBBP/CREB-binding protein/E1A-binding protein P300 (EP300) or P300/CBP-associated factor (PCAF, aka KAT2B) and General control of amino acid synthesis protein 5 (GCN5, aka KAT2A), together with chromatin-remodeling complexes to activate transcription [22,23]. Strawberry Notch (Sno), another modulator of the pathway whose role is still unclear, appears to act downstream to disrupt the CSL repression complex [24].

#### 2.3.2. Post-Translational Modifications

Notch has many types of post-translational modifications (PTMs) that affect its function. The post-translational modifications of Notch are categorized as extracellular and intracellular. The Notch extracellular domain (NECD) is predominantly modified by glycosylation [N-glycosylation and O-glycosylation (O-fucose, O-glucose, and O-GlcNAc)], whereas the intracellular structural domains are predominantly modified by hydroxylation, phosphorylation, and ubiquitination. Ubiquitination of the intracellular structural domain regulates receptor trafficking and protein hydrolysis, whereas glycosylation of the NECD affects ligand binding and activation [25].

Glycosylation modifications of the NECD that affect Notch function have been reported. Notch and its ligands are modified by a protein O-fucosyltransferase1 (Pofut1 in mammals, Ofut1 in flies). Reducing O-fucose levels by decreasing Ofut1 expression in *Drosophila* through RNAi inhibits Notch signaling during *Drosophila* wing development, and these are Fringe-dependent and Fringe-independent processes [26]. Mouse embryos lacking the Pofut1 protein have severe defects in mid-gestation mortality and neurogenesis [27]. Pofut1 affects Notch folding localization and ligand binding. O-glucosylation is also essential for Notch activity. The enzymes that mediate the addition of O-linked glucose to EGF repeats are Rumi (in *Drosophila*) and Poglut1 (O-glucosyltransferase 1 in mammals), and the knockout of Rumi mutants in flies and Poglut1 in mice both produce Notch-like phenotypes. O-glycosylation is involved in S2 cleavage and affects Notch receptor hydrolysis after ligand binding. It has been reported that O-GalNAc glycan modifies Notch outside the EGF repeat sequence near the S2 cleavage site [28]. The role of O-GlcNAcylation on Notch is incompletely understood.

The magnitude and duration of the Notch response depends on post-translational modifications of the activated NICD. Factor-inhibiting hypoxia-induced factors (HIFs) (FIH) have been shown to hydroxylate the NICD and mediate oxygen-dependent Notch signaling. Hydroxylation-deficient NICD1 function is impaired in Notch-dependent neurogenesis in *Danio rerio* (*D. rerio*), and the NICD1 is more ubiquitinated [29]. Phosphorylation involving CDK1 and CDK2 leads to the FBXW7-mediated degradation of the NICD and affects both the clock period and somite size [30]. Phosphorylation is involved in the cleavage of the S2 site. Many kinases can also directly control transcriptional activity; for example the NLK-phosphorylated ANK structural domain reduces transcriptional activity by interfering with ternary complex formation [25]. Ubiquitination modifications of Notch can be divided into two categories: ubiquitination at the receptor level and ubiquitination at the ligand level. E3 ligases of the RING and HECT families are involved in ubiquitination, mainly determining the half-life and signaling potential of Notch. E3 ligases, namely suppressor of Deltex [Su(dx)] and Nedd4, primarily regulate vesicular transport and the stability of Notch. The ubiquitination of Notch can be subdivided into mono- and poly-ubiquitination, with mono-ubiquitination mediating the normal vesicular transport of Notch, whereas poly-ubiquitination leads to Notch degradation by the proteasome. Enzymes involved in ligand ubiquitination include Neuralized (Neur) and Mindbomb (Mib); deficiency of either of these enzymes does not trigger the Notch signaling cascade and, deficiency of Mib1 is lethal to mouse embryos [31].

Other post-translational modifications regarding Notch include acetylation and methylation. Several acetyltransferases, such as PCAF and GCN5, have been reported to directly acetylate Notch ICDs. However, the histone deacetylases Sirtuin 1 (SIRT1) and histone deacetylase 1(HDAC1) deacetylate it. Acetylation attenuates the ubiquitination and degradation of the NICD [32]. Similar phenotypes have been reported to arise from Sirt1 mutants and Notch deletions in *Drosophila*. In addition to post-translational modifications of other components of the Notch signaling pathway, which also affect Notch signaling activity and function, individual components of the ternary CSL-NICD-MAML complex are also acetylated to regulate Notch transcriptional activity through effects on chromatin. Methylation of the NICD occurs predominantly in the nucleus and is performed on five conserved arginine residues within the C-terminal transactivation domain (TAD). Methylation stimulates Notch transcriptional activity on the one hand and increases ubiquitin-mediated degradation of the NICD on the other [33]. There is growing evidence that epigenetic modification mechanisms regulate Notch transactivation activity. Evidence suggests that a series of non-coding RNAs regulate Notch signaling, such as microRNA (miR)-26 family members miR-26a and miR-26b, which act against epithelial–mesenchymal transition (EMT) by directly targeting Jagged-1 and inhibiting Jagged-1/Notch signaling [34]. In addition, the signaling activity of Notch is also regulated by endocytosis, and the endocytosis of Notch is associated with ubiquitin E3 ligases. The two E3 ligases that mediate the endocytic transport of Notch are Su(dx) (in fruit fly)/Itch (in mammals) and Nedd4. Itch and Nedd4 act as inhibitors of Deltex (DTX), but DTX acts as an activator of non-canonical Notch signaling by ubiquitinating the ICD of Notch and facilitating the process of endocytosis [35,36]. Although the biochemical details of the non-canonical Notch pathway remain to be elucidated, the existence of this pathway, which is independent of CSL or γ-secretase activity, indicates the complexity of the modulation of Notch signaling activity [37]. We have not further summarized the non-classical Notch pathway.

## 3. Functional Roles of Notch Signaling Pathway Members in Invertebrates

The functions of the canonical Notch signaling pathway have been most extensively studied in vertebrates and classical invertebrate models such as *D. melanogaster* and *C. elegans*. The expression profiles and roles of the core components of the Notch cascade have been summarized in many reviews (reviewed in [37,38,39]). However, knowledge about their expression and functions in other invertebrates, especially in aquatic organisms, is still limited. Here, we mainly discuss the available information on Notch signaling pathway members in the regulation of neurogenesis and regeneration in invertebrates (Table 1). For the sake of brevity, we refer to most species by their generic names, as taxonomic revisions at the species level are common.

### 3.1. Choanozoa: Monosiga brevicollis (M. brevicollis)

Choanoflagellates are widely distributed eukaryotic microorganisms that are found in marine and freshwater environments. Choanoflagellates are evolutionarily important because they are close relatives of metazoans [134]. More than 125 species of flagellates have been identified, all of which have unicellular life history stages [135]. Previous studies have provided more insight into the molecular biology of the choanoflagellate *M. brevicollis* through genome sequencing. Genomic analysis of *M. brevicollis* revealed complete deletion of many signaling pathways but the retention of cassettes of protein domains of the Notch receptor [(EGF, NL, and ANK (ankyrin repeats)] [10,136]. No homologues for Delta were found in M. brevicollis, but homologues for Presenilin were found. In addition, genes partially homologous to Furin and TACE were identified [136]. Nevertheless, the genome of *M. brevicollis* does provide insights into the evolution of the Notch signaling pathway.

### 3.2. Porifera/Spongia

Sponges (Porifera), called Porifera because they are porous or ‘pore bearing’, are likely to be the earliest branching animal phylum [137]. Nowadays, sponges are considered unquestionably metazoans, they are a more ancient phyletic lineage than the eumetazoans (cnidarians and bilaterians), and they lack neurons, limbs, eyes, and segments. However, despite their apparent anatomical simplicity, many components of the major signaling pathways that modulate neurogenesis in bilaterians are conserved in sponges; for example, the sponges *Oscarella carmela* (*O. carmela*), *Oopsacas minuta* (*O. minuta*), *Aphrocallistes vastus* (*A. vastus*), and *Amphimdeon queenslandica* (*A. queenslandica*) all express the core components of Notch [40,41]. As the key role of Notch signaling in Eumetazoa is the specification, determination, and differentiation of neuronal cell types [138], the presence of Notch signaling components in sponges without a true nervous system suggests that the Notch signaling pathway predates the emergence of the nervous system.

In glass sponges (*O.minuta* and *A.vastus*), there are one Furin, two ADAM10/17 proteins, and the four γ-secretase subunits which are required for S1, S2, and S3 cleavages, respectively. They all show a similar domain composition to their human orthologs. Although no MAML protein was retrieved, the presence of a unique Delta protein (*OmDelta* and *AvDelta*), true Notch receptor (*OmNotch* and *AvNotch*), and the CSL/SuH transcription factor indicated that Notch activation and signal transduction may occur in glass sponges. However, functional studies are required to prove ligand–receptor interactions in poriferans [40].

In the porifera *A. queenslandica*, there are five membrane-bound Delta ligands (*AmqDelta1-5*), one Notch receptor (*AmqNotch*), and other orthologs such as *AmqbHLH1* (Table 1), which regulate canonical Notch signaling events. *AmqDelta1-5* possess TM, MNLL, EGF (except *AmqDelta4*), and DSL domains, and these are overlappingly expressed throughout the embryonic development of *A. queenslandica* and persist into the larval stage [41]. The N-terminal valine of the TM structural domain in all *AmqDelta* may be a cleavage site for γ-secretase [139]. *AmqNotch* is expressed in both embryonic and larval stages, while *AmqbHLH1* is expressed in a field of subepithelial cells during Amphimedon development. *AmqbHLH1* is homologous to all the atonal-related bHLH genes found in bilaterians [42], and *AmqbHLH1* has structural and functional properties that can be found in bilaterian pro-neural proteins and are required to promote neural development.

There is much evidence to suggest that the Notch signaling pathway may be involved in sponge neurodevelopment. First, many of the cells expressing Delta in *A. queenslandica* may perform sensory functions in larvae. Second, larval flask cells expressing *AmqDelta 3* and 4 are morphologically most akin to sensory cells in eumetazoan [140,141]. Third, globular cells expressing *AmqDelta1* and anterior polar cells expressing *AmqDelta1* and 4 also express the neurogenic transcription factor bHLH [142], and these homologs of bHLH are involved in neural development. Additionally, ciliated cells expressing *AmqDelta4* together with a homologous cassette gene LIM with neural function were the only cells confirmed to have sensory function [143]. These suggest a common ancestry between porifera sensory cells and metazoan neurons [41,138].

### 3.3. Cnidaria

Cnidarians, also known as coelenterates, are an early-branching basal metazoan group consisting of four classes: Hydrozoa; Scyphozoa; Anthozoa; and Cubozoa. They are mostly marine animals (e.g., corals, anemones, hydroids, and jellyfish) and tend to share several similarities, including a relatively simple organization around a central body cavity [144]. The cnidarians have a high capacity for tissue plasticity and regeneration such as the regeneration of the head of *Hydra*, the regeneration of the whole-body axis of *Nematostella vectensis* (*N. vectensis*), the regeneration of tissues in stony corals, and the regeneration of limbs in moon jellyfish [145,146,147,148]. Cnidarians do not have a centralized nervous system but possess neural cells. The nervous system of cnidarians comprises endodermal and ectodermal nerve nets. The neural cell types found in cnidarians include sensory cells, ganglion cells, and cnidocytes (a cnidarian-specific mechanosensory cell) [149,150]. Cnidarians have been shown to have considerable genomic complexity and possess nearly complete repertoires of all major metazoan signaling pathways [151]. It is supported that Notch pathway components in cnidarians may help to better understand the origin of Notch pathway involvement in development, particularly neurodevelopment.

#### 3.3.1. Hydrozoa

Hydrozoa [species, *Hydra vulgaris* (*H. vulgaris*), *Hydra oligactis* (*H. oligactis*), *H. braueri* (*H. braueri*), *Hydra viridissima* (*H. viridissima*), and *Hydractinia echinata* (*H. echinata*)] are the sister group to all bilaterians, and they possesses one of the most “primitive” known nervous systems in the form of a diffusely distributed nerve net [152,153]. The regenerative capacity of Hydra is extraordinary, with them being able to regenerate body parts but also regenerate entire animals from a clump of dissociated tissues.

Notch pathway components that have been reported in different *Hydra* species include *HyJagged*, *HvNotch*, *HvSu(H)*, *Hvpen-2*, and *HyHes-1*. The putative Notch ligand *HyJagged* is a transmembrane protein present in all cell types of adult *Hydra* and upregulated at the boundary between bud and parent [43]. *HvNotch* (*Notch-a* and *Notch-b*) expression was observed in both the ectodermal and the endodermal cell layers. The expression of the *HvNotch* target gene *HyHes* mirrored the *HvNotch* pattern during regeneration. It was upregulated in regenerating heads after 24 h and was later restricted to the emerging tentacle buds. The expression of *HyHes* during regeneration is controlled by Notch signaling [44].

Previous studies have shown the inhibition of hydroid head regeneration using DAPT, and the regenerating tissue has been unable to re-establish an oral organizer and evenly spaced tentacles. In addition to affecting head regeneration, DAPT treatment of *Hydra* polyps causes distinct differentiation defects in their nematoblast (which gives rise to the cnidarian-specific sensory cells, nematocytes) and germ cell lineages, and impairs boundary formation at both the parent–bud and body column–tentacle boundaries [44]. The above-mentioned phenotypes caused by DAPT are due to the inhibition of the NICD translocation by DAPT. Thus, the Notch signaling pathway is required for *Hydra* head regeneration [44].

The reappearance of the nervous system during *Hydra* regeneration has also been investigated, and the potential to regenerate a nervous network in *Hydra* has been exploited through nervous system transplantation studies [154]. However, inhibition of the Notch signaling pathway affected *Hydra* regeneration but did not inhibit neural development, at least with respect to RFamide^+^ and GLWamide^+^ neurons [45]. The role of Notch signaling in the development of the *Hydra* neural network remains to be elucidated in depth.

#### 3.3.2. Anthozoa: *N. vectensis*

Sea anemones are apparently primitive animals belonging to the invertebrate order Actiniaria (class Anthozoa, phylum Cnidaria). *N. vectensis* are highly regenerative sea anemones, capable of regenerating lost/missing body parts in less than 1 week even from small isolated fragments, suggesting that it is a good choice as a model system to compare development and regeneration, especially for understanding muscle development and neurogenesis (cnidarian muscles are closely linked to the nervous system) [155,156].

The nervous system of *N. vectensis* is a diffuse nerve network containing both ectodermal sensory and effector cells and endodermal multipolar ganglion cells. This network consists of several distinct neural territories along the oral–aboral axis, including the pharyngeal and oral nerve rings and the larval apical tuft. Neural specification in *N. vectensis* appears to occur by a mechanism independent of that in the classic cnidarian model *Hydra* [150].

In *N. vectensis*, homologous genes for mammalian Notch signaling components include *NvDelta/NvJagged*, *NvNotch*, *NvSuH*, and *NvHes1-4*. Notch signaling components are expressed during gastrulation and planula and polyp formation in *N. vectensis* in overlapping domains in both ectodermal and endodermal tissues. Heather Marlow et al. used the γ-secretase inhibitor DAPT to block the internalization and subsequent activity of the NICD during *N. vectensis* development. The results show that in DAPT-treated embryos, in which pharyngeal growth is stunted, tentacle formation is defective, mesentery formation fails to occur, and endoderm morphology is disrupted, the endoderm is stratified and disorganized, and cnidocyte sting cells are completely absent compared to control embryos. In addition, blocking the Notch signal can lead to an upregulation of neural marker genes and the emergence of a ‘neurogenic’ phenotype. Knockdown of Su(H) transcription factor expression levels is similar to the phenotype produced by the inhibition of Notch receptor cleavage [46].

Notch signaling regulates neural development in *N. vectensis* by acting in concert with *NvAth*, *NvAshA*, and *NvSoxB*. Complementary functions of the SoxB gene and Notch signaling act as positive and negative regulators of *N. vectensis* neurogenesis, respectively. Notch activity suppresses neurogenesis by repressing *NvAshA* expression [47]. It remains inconclusive whether Notch signaling in nematode neurogenesis occurs through a Hes-dependent mechanism. Overexpression of the *NvHes* genes did not alter *NvAshA* expression levels. Further studies are required to determine which of these Hes genes, or combination thereof, is responsible for the cnidocyte phenotype in *N. vectensis*. The common requirement for Notch signaling in the development of both cnidocytes and neurons further supports the hypothesis that cnidocytes and neurons share a common origin as multifunctional sensory cells. Inhibition of Notch signaling blocks regeneration of the anemone head [48]. Thus, inhibition of Notch signaling blocks *Nematostella*’s regeneration and neurodevelopment.

Coral is another marine organism belonging to the class of the Anthozoa (phylum Cnidaria), with an external or internal skeleton as its characteristic feature. Transcriptome-wide gene profiles differ significantly between different parts of the coral colony as well as between species. Components of the Notch signaling pathway were obtained by transcriptome analysis of *Acropora* and *Orbicella faveolata* (*O. faveolata*). Notch signaling molecules in Acropora [the staghorn coral, *Acropora cervicornis* (*A. cervicornis*) and the elkhorn coral, *Acropora palmata* (*A. palmata*)] including Delta/Delta-like, Jagged, Notch, Hairy/E(Spl), Su(H), E3 ubiquitin ligase MIB and Numb [49]. While components of the Notch signaling pathway in *O. faveolata* transcriptome data include Delta-like ligand (DLL), protein jagged, metalloproteinase domain-containing protein 17 (ADA), neurogenic locus NOTCH, E3 ubiquitin-protein ligase DTX1, PEN, NICA, PSN, APH, E1A/CREB-binding protein (EP300), RBPJL, Hairless, HES among others [50]. Analysis of the data shows that Notch signaling was enriched at the branch tips of corals, suggesting that Notch is involved in Acropora growth and that Notch signaling in *O. faveolata* may be involved in innate immune defense. Notch signaling has been shown to be involved in development, regeneration, and neurogenesis in cnidarians but has not been functionally explored in corals and is currently hypothesized to be involved in coral neurogenesis and tissue regeneration.

### 3.4. Platyhelminthes

The phylum Platyhelminthes (flatworms) is usually a group of soft-bodied and much-flattened invertebrates, consisting of four classes: Trematoda (flukes), Cestoda (tapeworms), Turbellaria (planarians), and Monogenea [157]. They are triploblastic (three embryonic layers: endoderm, mesoderm, and ectoderm), acoelomate, and bilaterally symmetric. Usually, they possess a digestive system, a primitive excretory system (protonephridia), a reproductive system (sexual reproduction, hermaphrodite), and an apparently primitive but well-organized central nervous system (consisting of an anteriorly located brain connected to a peripheral nervous system and a variety of sensory organs) [158,159,160]. However, platyhelminthes have captured the imagination of biologists for centuries because planarians are unparalleled experimental models for studying stem cell regulation and tissue regeneration. The molecular architecture of the Notch signaling pathway is well conserved in flatworms and this pathway is involved in flatworm regeneration, particularly of the nervous system [51,52].

#### 3.4.1. Turbellaria: *Dugesia japonica* (*D. japonica*) and *Schmidtea mediterranea* (*S. mediterranea*)

Most turbellarians (planarians) are exclusively free-living forms and entirely carnivorous. They have a bilateral symmetric central nervous system (CNS) that consists of an archaic brain and one or several pairs of longitudinal nerve cords [158,161]. The discovery of the regenerative abilities of turbellarians goes back over 200 years, when they were found capable of regenerating the entire body [162]. Indeed, Morgan has previously reported that a planarian can regenerate from tissue fragments as small as 1/279th the size of an intact worm This excellent regenerative ability relies on a population of pluripotent stem cells called neoblasts [159]. In addition, their strong ability to regenerate ensures the success of their asexual reproduction strategy.

The most studied planarians worldwide are *D. japonica* and *S. mediterranea*. *DjNotch1-6* have been identified in the *D. japonica*, while at least three Notch genes (*SmedNotch1*, *SmedNotch2*, *SmedNotch4*) have been found in *S. mediterranea* [53]. In *D. japonica*, *DjNotch1-6* are widely and strongly expressed in the parenchymal tissues and the blastema site, with particularly strong expression at both body margins and midline in tail segments. However, only *DjNotch1* and *DjNotch2* possess classical EGF, LNR, ANK repeats, and a transmembrane domain. The Notch signaling pathway has been shown to be involved in tissue regeneration and neurodevelopment in *D. japonica* [51], as DAPT treatment (100 nM for 10 days) significantly decreased the expression of *DjNotch1*, *DjNotch2*, *DjCSL,* and *DjHes* and caused many regeneration defects, including multi-eye spot deformities (head fragments) or cyclopia (single eye of tail fragments), black patches, asymmetric or small heads, abnormal neurodevelopment, and midline patterning. In addition to this, DAPT-treated *Dugesia japonica* showed abnormal motor retardation and elevated levels of cellular accretion and apoptosis [51]. Many of these various defects were also found in animals treated with RNAi against Notch genes (*DjNotch1-5*). Similar phenotypic defects have been shown to be produced by knocking down Notch signaling components (Notch-2, Delta-3 and Rbpj) or miR-124 in *S. mediterranea*. The Notch signaling component is a predicted target of miR-124, and miR-124 may regulate the midline Slit-1-expression via canonical Notch signaling [53]. Taken together, the data suggest that Notch signaling is critical for the regeneration of planarians, especially the nervous system.

Forty-four planarian bHLH homologs, such as Atoh, Coe, Fer3l-1, Hesl-3 by, and Sim et al., have been identified by genome-wide analysis [52]. They are expressed in discrete neural populations throughout the brain and in regenerating tissues. In addition, Coe, Hesl-3, and Sim were each co-expressed in cholinergic neurons, while Fer3l-1, Hesl-3, and Sim were detected in cells distributed throughout the mesenchyme. RNAi screening revealed that Coe, Hesl-3, or Sim resulted in significant defects in brain regeneration, and a loss of expression of genes unique to distinct neuronal subtypes. Coe (RNAi) regenerates displayed photoreceptors with abnormal morphology and smaller cephalic ganglia, which failed to form anterior commissures. In Hesl-3 (RNAi) regenerates, the CNS was abnormally patterned, showing abnormal brain morphology with single or ectopic eye spots during regeneration. Sim (RNAi) animals regenerated photoreceptors with reduced pigmentation and displayed a reduced brain neuropil density. Knockdown of Arnt or Sim results in similar regeneration defects, and the two genes interact with each other. In addition to this, RNAi knockdown of Coe, Hesl-3, or Sim resulted in neuronal subtype-specific gene expression deletions. These data show that these bHLH genes are required to generate new neurons in uninjured and regenerating animals [52]. The above studies confirm that the Notch signaling pathway influences the regeneration of planarian, especially the nervous system. However, the Notch signaling pathway components and cofactors are still not fully investigated in planarian.

#### 3.4.2. Trematoda: *Schistosoma mansoni* (*S. mansoni*)

Schistosomiasis, also known as bilharzia, is a disease caused by parasitic worms called trematodes (flukes) [163]. The three main species that infect humans are *Schistosoma haematobium* (*S. haematobium*), *Schistosoma japonicum* (*S. japonicum*), and *S. mansoni* [164]. *S. mansoni* has a complex life cycle. It requires two hosts and several life stages to complete its developmental cycle. The eggs shed by the adult worms represent an important stage in the life cycle and are a critical step in transmitting the disease [165]. The Notch pathway is required for oogenesis and embryogenesis in *S. mansoni*, as treatment with low concentrations of DAPT (<10 μm) does not affect the viability or motor activity of adult worms but reduces the production of phenotypically normal eggs and inhibits egg development in vitro [54].

Notch signaling components that have been identified in *S. mansoni* include *SmJagged/SmSerrate*, *SmNotch*, *SmSu(H)*, *SmHes/Hairy*, co-repressors (*SmSmart* and *SmGroucho*), co-activator *SmSkip*, γ-secretases (*smPresenilin*, *SmNicastrin*, *SmAph-1*, and *SmPen-2*), *SmFurin*, metalloproteases (*SmAdam 17* and *SmKuzbanian*), and putative regulated genes (Notchless, Numb, Dishevelled, and WWP1) of the Notch pathway. The identified Notch signaling components are present in all the life cycles of *S. mansoni*. However, in the schistosomula stage, the expression level of *SmHes* was significantly decreased compared to that in eggs. *SmNotch*, *SmSu(H)*, *SmHes*, and *SmAph-1* transcripts in the cercariae were also significantly downregulated compared to their respective levels in eggs. In contrast, the transcripts of *SmNicastrin* and *SmPen-2* showed no expression differences. It follows that most of the Notch signaling pathway components and associated regulatory molecules that occur in higher vertebrates are present in this species. However, the number of their domains differ. For example, only two of the eight predicted sequences for the Notch receptor in *S. mansoni* exhibited the typical architectures (i.e., extracellular region with repeats of the EGF and NRL domains, transmembrane region, with the intracellular portion bearing repeated ANK domains) of Notch receptors found in other organisms, and these two Notch receptors, like the Notch receptors in lower invertebrates such as *A. queenslandica* and *Hydra vulgaris* (*H. vulgari*), all have a short extracellular region (fewer EGF repeats) compared to those found in vertebrate species. The identified Notch signaling components are present in all life cycles of *S. mansoni*, although the level of expression of some genes changed at each stage. Whether the Notch signaling pathway is involved in *S. mansoni* neurogenesis remains to be investigated [54].

Previous studies have shown that *S. mansoni* possesses a complex neurological and in vitro regeneration case, and nowadays there is still no functional study of Notch signaling involved in both [166,167].

#### 3.4.3. Cestoda: *Echinococcus granulosus* (*E. granulosus*)

Cystic echinococcosis and alveolar echinococcosis (*E. granulosis*), the diseases caused by the metacestode larval stage of the tapeworms *E. granulosus* and *Echinococcus multilocularis* (*E. multilocularis*), respectively, are a global zoonosis [168]. As the intermediate hosts, humans are usually infected by the oral ingestion of eggs containing oncospheres. Once the eggs mature into hydatid cysts filled with fluid and protoscoleces, the protoscoleces can infect both the definitive and intermediate host.

Whole genome sequencing as well as proteomic and transcriptional studies have identified genes and proteins that are differentially expressed between the different life stages and cyst components of *E. granulosus*. These include the Notch components (Delta/jagged, Notch, PS1, Aph-1, Pen-2, Su(H), ADAM 17-like protease, etc.) [55]. The Notch gene was expressed at all developmental stages of *E. granulosus*, although the expression levels at different stages are different, with Notch significantly upregulated only in the germinal layer and vesicle/microcyst formation, suggesting a probable role of Notch gene products in the mitotic cell division and proliferation of *E. granulosus* [56]. Interestingly, bioinformatic analysis revealed that Notch components are enriched as alternative splicing (AS) genes in response to environmental information processing, and that extracellular vesicle (EV)-hosted miRNA are involved in regulating *E. granulosus* encystation by targeting Notch pathway mRNA [57,58]. Whether the Notch pathway is involved in neural development and regeneration in *E. granulosus* needs to be further investigated.

### 3.5. Nemathelminthes, Nematoda: Caenorhabdits elegans (C. elegans)

The nematode *C. elegans* is a genetically tractable model for studying regenerative responses in neurons, e.g., damaged axons can in some cases regenerate, restoring function to the nervous system after injury or disease [169]. The nervous system of *C. elegans* consists of 302 neurons, 50 glial cells derived from neuronal/epithelial precursors, and six glial cells of mesodermal origin [170]. Neurons are specified by a combination of transcription factors inherited from ancestral cells and signaling between neighboring cells (in particular Wnt and Notch signaling) [171]. The importance of the Notch signaling pathway in *C. elegans* neurodevelopment and regeneration is well summarized in many reviews [172,173,174], and here we will only briefly introduce the function of Notch components in *C. elegans*.

The core components of Notch in *C. elegans* include the following: four ligands Lag-2 (Sel-3), Apx-1, Arg-1, Dsl-1 [59,60,61,62]; two receptors Lin-12 and Glp-1 (Notch1-4, in mammals) [63]; nuclear complexes Lag-1 (CSL and RBPJ, in mammals) and Sel-8/Lag-3 (Mastermind, in mammals); γ-secretase components Sel-12/Hop-1 (presenilin1/2, in mammals), Aph-1, Aph-2 (nicastrin, in mammals), Pen-2 [175,176,177,178,179]; and target genes Lin-12, Mir-61/250, Lip-1, Lst1-4, Fbf-1, Pkg-1/Egl-4 (Hes, in mammals) [64,65,66].

In a 4-cell *C. elegans* embryo, two sister blastomeres called ABa and ABp have equivalent developmental potential but different cell fates because ABp receives a signal from the posterior-most blastomere P2 and this P2/ABp interaction depends on Glp-1 receptor and Apx-1 ligand, which suggests that Lin-12 and Glp-1 are required for the regulation of cellular interactions during development in *C. elegans* [180]. Double mutants lacking zygotic Lin-12 and Glp-1 activity die as L1 larvae with a variety of discrete defects [181], demonstrating that Lin-12 and Glp-1 are functionally redundant despite their divergent sequence, which can be validated because Glp-1 can replace Lin-12 in cell fate decisions [182]. Aph-2 is expressed in all early blastomeres and is essential for Notch signaling in early embryogenesis [183]. Its mutations cause embryonic lethality, anterior pharyngeal defects, and oviposition defects (Egl phenotype) in *C. elegans* [177,183,184]. Aph-1 mutant embryos lack an anterior pharynx but can develop a relatively normal posterior pharynx [185]. Removal of other Notch components such as Glp-1, Lag-1, or the two presenilin proteins SEL-12 and HOP-1 results in an Aph-mutant phenotype [175,176,186,187], further reflecting the partially redundant effects of Notch pathway signaling.

Glp-1 and Lin-12 facilitate signaling by recruiting Lag-3 to target promoters, where Lag-3 functions as a transcriptional activator, and their main function is to link Lag-3 and Lag-1 to form a ternary complex at the target gene promoter. The reduced expression of Lag-3 by RNAi shows that C. elegans embryos lack anterior pharynx and die within the eggshell. The post-embryonically effect was examined by immersing early L1 larvae in Lag-3 dsRNA, resulting in adults that were usually sterile and had prominent vulvae, and sterility was due to the failure of germline appreciation [188], a phenotype typical of Glp-1-null mutants [189]. In addition, the prominent vulva phenotype was similar to that of the Lin-2 mutant [190]. The effect of Lag-3 (RNAi) on Glp-1 and Lin-12 gain-of-function mutants was found to be that most Glp-1 (oz112 gf) are sterile and make tumorous germ lines, which was similar for Lin-12. All Lin-12 (n137gf) mutants possess multiple pseudo-vulvae, the Muv phenotype, and do not possess an anchor cell. Lag-3 was also shown to correspond to the previously described locus Self-8 and was associated with the Lag phenotype in RNAi experiments. Thus, both Glp-1 (gf) and Lin-12 (gf) receptors depend on Lag-3 activity for signaling [188].

The Lag-2 ligand is expressed in IL2 neurons and activates the typical Glp-1 Notch signaling pathway. Roles for Lin-12 signaling in the nervous system of adult Hidradenitis elegans cryptic nematodes include the following: first, altering Lin-12 activity increases spontaneous reversal during locomotion; second, Lin-12 is expressed in larval RIG neurons and acts in a subset of Glr-1-expressing neurons to regulate reversal; third, Lin-12, Lag-2, and Lag-1 may act together in the nervous system to regulate reversal [172]. Thus, the Lin-12 Notch receptor is a potent inhibitor of regeneration in *C. elegans* motor neurons [173]. Both the metalloprotease Sup-17/ADAM10 and presenilin are required for Notch to inhibit regeneration, and overexpression of the NICD is sufficient to inhibit regeneration [169].

Genes with aggregated Lag-1 binding sites (LBSs) are potential direct targets of Lin-12 [191]. Egl-4 encodes a cGMP-dependent protein kinase (PKG) that is expressed in many head neurons; it is not only involved in the octanol response of the *C. elegans* but also regulates its body size and behavioral state [65,192]. Lst1-4 are genes with clustered LBSs and are direct targets of Lin-12 Notch signaling in the vulval cell fate specification of *C. elegans*, in which Lst-1 lacks a mammalian direct homologue [66]. Lst1-4 is expressed in VPCs (the six vulval precursor cells). Lst-1 is expressed in neurons in the head of *C. elegans*, including octanol-sensing AWB neurons [193]. Animals lacking Lst-1 have a defective octanol response [65]. Depletion of Lst2-4 caused ectopic vulval induction [66]. Egl-4 encodes a PKG that is expressed in many head neurons and regulates body size and behavioral state in *C. elegans* [192,194,195,196]. Egl-4 is involved in the octanol reaction [65]. These findings suggest that Notch signaling core components are involved not only in the developmental and behavioral norms of *C. elegans* but also in neural development, a behavior that is consistent with mammals.

### 3.6. Annelida

The phylum Annelida is a group of bilaterally symmetrical animals with a body cavity (or coelom) and highly adaptive segmentation, usually segmented by shallow but visible rings called annuli that encircle their bodies. Earthworms (Oligochaeta), leeches (Hirudinea), and marine worms (Polychaeta, which are divided into tube-dwelling, sedentary, or free-moving forms) are three classes of annelids. Annelida’s central nervous system consists of two small cerebral ganglia connected by a dorsal commissure, a ventral nerve mass, and a pair of long circum-esophageal connectives joining the former to the latter [197]. The involvement of Notch signaling in the neural development of annelids has been reported. The ability to regenerate extensive portions of the body is widespread among the Annelida phylum, and this group includes some of the most highly regenerative animals known [198]. Among them are *Enchytraeus fragmentosus* (*E. fragmentosus*) and *Stylaria lacustris* (*S. lacustris*), which regenerate the nervous system; Earthworm, *Eisenia Andrei* (*E. Andrei*) which regenerates segments; and *Autolitus pictus* (*A. pictus*), which regenerates the pharynx [199,200,201]. Notch signaling is involved in a large range of developmental processes and has been functionally implicated in body plan segmentation in two of the three diverse segmented taxa: the vertebrates and arthropods.

#### 3.6.1. Earthworms: *Perionyx excavates* (*P. excavatus*)

The earthworm *P. excavatus* is a species with a high capacity for bidirectional regeneration. This species is able to regenerate its anterior parts and rebuild its reproductive organs after amputation. These properties make it a good model for bidirectional regeneration. Using expressed sequence tags, *Pex-Notch*, *Pex-DeltaD,* and *Pex-Serrate* were found in the gene expression profiles associated with anterior regeneration in the earthworm *P. excavatus.* Among other things, Notch expression is upregulated during the anterior regeneration of *P. excavatus* [67]. In addition, *Pex-Hes1A*, *Pex-Hes1B*, *Pex-Hes4A*, and *Pex-Hey* showed variable expressions during bidirectional regeneration [68]. *Pex-Hes1A* and *Pex-Hes1B* were not expressed in intact tissues, whereas *Pex-Hes4A* was expressed and highly expressed in regenerated head and tail tissues. *Pex-Hes1B* was only weakly expressed early in bidirectional regeneration. *Pex-Hey* is present in intact tissues and throughout bidirectional regeneration. It is predicted that very early activation of a Notch-like receptor may be a regeneration-inducing pathway that precedes the regeneration process. Further studies, such as knockdown experiments, are needed to elucidate the specific functions of these genes. Despite studies depicting the bilobed mass brain (located above the pharynx in the third somite of earthworms), its associated sensory nerves, and other neural structures, there have been few studies on the neurodevelopmental function of Notch signaling in earthworms. Following the completion of genome assembly and analysis in the earthworm *Amynthas cortici* (*A. cortici*) [69], further studies can be undertaken to analyze Notch regulation in neural development or regeneration.

#### 3.6.2. Polychaeta: *Platynereis dumerilii* (*P. dumerilii*)

Platynereis is an important lophotrochozoan model species in evolutionary and developmental biology. The major wild-type strains of Platynereis have been continuously bred in captivity for more than 60 years, and several highly inbred strains, some stable transgenic, and mutant strains are available and easily reared at low cost in inland laboratories. Among these strains, the sandworm *P. dumerilii* is a very useful invertebrate for studying the brain of the past and regeneration, as live imaging and the manipulation of neurons are possible in these translucent animals, and *P. dumerili* has the ability to regenerate caudal segments after the posterior end is amputated from the adult worm [70].

The Notch signaling component in *P. dumerilii* consists of two classical ligands *Pdu-Delta* (two splice variants: *Pdu-Delta^tv1^* and *Pdu*-*Delta^tv2^*) and *Pdu-Jagged*, the receptor *Pdu-Notch*, the transcription factor *Pdu-Su(H)*, the γ-secretase complex component *Pdu-Presenilin,* and the downstream genes *Pdu-Hes1-15* [Hairy/E(Spl)] [71,72]. Some of these proteins are highly conserved in their domain arrangement and are likely to be the ancestral in the bilaterian lineage. By using whole-mount in situ hybridization (WMISH), it was indicated that the expression of Notch core components [*Pdu-Notch*, *Pdu-Delta*, *Pdu-Jagged* and *Pdu-Su(H)*] was distributed across three major structures in larvae: the chaetal sacs, and some brain cells including the apical organ and the stomodeum. Specifically, *Pdu-Notch*, *Pdu-Delta*, and *Pdu-Su(H)* are expressed in the chaetal sacs, and all four genes are expressed in different populations of brain cells and in the stomodeum at different stages. However, during the most important stage of neurogenesis, the expression of Notch components is very limited, and treatment with chemical inhibitors confirmed that Notch does not play a major role in general larval neurogenesis but is important for the formation of chaetal sacs. Nevertheless, a slight increase in the number of cholinergic neurons in the ventral nerve cord (VNC) after drug treatment suggests that the effects of the Notch pathway on peripheral neuron differentiation cannot be ruled out.

In addition, numerous orthologs of the major vertebrate neural bHLH genes (achaete–scute, neurogenin, atonal, olig, neurod, twist and Hes/Hey), which have both pro-neural and neuronal specification functions, are also found in *Platynereis* [73]. Among them, the atonal- and achaete–scute-related bHLH genes are expressed during the formation of the trunk nervous system, and all 13 Hes/Hey-related genes are expressed in cells that are crucial for the formation of the central and peripheral nervous system (PNS), e.g., Hes12 and Hes13 are present in the presumptive VNC during larval neurogenesis. This expression pattern of the Hes/Hey gene suggests its involvement in neurological development, but also reminds us of the Notch-independent functions of the Hes family. In some contexts in vertebrates, twist is highly correlated with Hey/Hes or is induced by Notch signaling. The relationship has not been reported in detail in *P. dumerilii*, but *Pdu-Twist* is involved in mesoderm formation during larval development, posterior growth, and caudal regeneration, as its mRNA is expressed in distinct domains within the posterior growth zone and in mesodermal anlagen and developing muscles [70,74].

Capitellidae *Capitella teleta* (*C. teletais*, previously known as *Capitella* sp. I) is another polychaete worm. *C. teletais* continues to produce segments throughout its life and exhibits strong posterior regeneration, including that of the ovaries [202]. The nervous system of *C. teleta* shares many features with other annelids, including a brain and a ladder-like ventral nerve cord with five connectives, reiterated commissures, and pairs of peripheral nerves [203]. Homologous genes for Notch components in *C. teletais* have been reported to include *CapI-Delta*, *CapI-Notch*, *CapI-Hes1-3,* and *CapI-Hesr1/CapI-Hesr2,* and these genes correlate with areas of high cell proliferation such as in the brain, foregut, and terminal growth zone [75,76]. During larval stages, *CapI-Notch*, *CapI-Delta*, *CapI-Hes2*, and *CapI-Hes3* have largely overlapping patterns of expression and their transcripts are initially detected in the broad ectodermal domains of future segments, as well as in the brain and foregut; later, they are all expressed in the terminal growth zone that generates post-metamorphic segments, with *CapI-Notch*, *CapI-Delta*, and *CapI-Hes2* transcripts being detected in the presumptive chaetal sacs. *CapI-Hes1* has a segmentally repeated pattern in a restricted region of the mesoderm in each presumptive segment, and it has a non-overlapping complementary expression pattern to that of *CapI-Notch* and *CapI-Delta*. In addition, *CapI-Hes2-3* and *CapI-Notch* are all expressed in the ganglia of the ventral nerve cord, but *CapI-Hes2-3* are also expressed in the CNS, whereas the expression of *CapI-Hes2* and *CapI-Hes3* in the juvenile growth zone does not overlap with that of *CapI-Notch* and *CapI-Delta*. The above data all suggest that three Hes genes may be regulated by Notch-dependent and Notch-independent mechanisms. The role of the extensive ectodermal expression pattern of Notch signaling homologs in *Capitella* sp. I in segment formation is not obvious. The expressions of *CapI-Delta*, *CapI-Notch*, *CapI-Hes2*, and *CapI-Hes3* in the brain of *Capitella* sp. I as well as in the ventral nerve cord speculate that Notch signaling is implicated in neurogenesis in *Capitella* sp. I. This speculation was answered in a study by Néva P Meyer, in which a region of high *CapI-Delta* expression roughly corresponded to the expression of the neuromarker gene *CapI-Ash1*, and *CapI-Notch* transcripts were detected at both the surface and the base of early brain neurogenesis at stage 4. This pattern of expression is consistent with brain nerve functioning [76]. In addition to this, it is speculated that Notch signaling may be involved in the development of chaetal sacs based on the location of the Notch signaling component’s expression in *Capitella* sp. I [75]. The expression pattern of the above Notch components identified in *Capitella* sp. I is not sufficient to account for a role in segmentation, and further discovery is needed to find out whether other components in the Notch have this function.

#### 3.6.3. Clitellata: *Helobdella robusta* (*H. robusta*)

The medicinal leech [*Hirudo medicinalis* (*H. medicinalis*)] is an important model system for studying the structure, function, development, regeneration, and repair of the nervous system [204]. The central nervous system of the leech consists of 32 bilateral neuromeres, with ~400 neurons arising from the ganglionic primordium of each segment [205]. *H. robusta* embryos express both Notch and Hes homologs (*Hro-Notch* and *Hro-Hes*, respectively) in various cells of the early embryo, including the teloblasts and blast cells of the posterior growth zone [77]. The use of DAPT alone did not result in detectable segmentation defects but did result in decreased levels of *Hro-Hes*, although the localization of *Hro-Hes* was not affected by treatment with DAPT. Segmentation was severely disrupted after combined treatment with DAPT and AS *Hro-hes* MO. In more severely affected lineages, cell proliferation and ganglion morphogenesis are affected throughout the injected lineage. The above functional assays show that Notch/Hes signaling may be involved in posterior elongation and segmentation in the lophotrochozoan species *H. robusta* [78].

### 3.7. Mollusca

Mollusks (or Molluscs) are soft-bodied invertebrates of the phylum Mollusca, many of which are wholly or partially enclosed in a calcium carbonate shell secreted by the mantle, a soft covering formed by the body wall [206]. Mollusks have evolved a well-developed nervous system with the emergence of cephalic, pleural, visceral, and pedal ganglia, and this nervous system, together with the endocrine system and a sophisticated immune system, forms a primitive neuroendocrine–immune (NEI) regulatory network [207]. Within Mollusca, cephalopods exhibit a particularly complex nervous system. The adult brain is formed from the fusion of several “typical” molluscan ganglia but it remains poorly understood how these ganglia emerge, migrate, and differentiate during embryogenesis [208]. Octopus and cephalopods are able to regenerate injured tissues. Furthermore, some molluscan species show remarkable brain regenerative ability and can achieve full functional recovery following injury. However, the molecular mechanisms of Notch signaling involved in neural development and organismal regeneration in Mollusca have been less studied [209,210].

#### 3.7.1. Gastropoda: *Ilyanassa obsoleta* (*I. obsoleta*)

*I. obsoleta* (*Eastern mud snail*), a host to many species of parasites, is a common inhabitant of salt marshes and can tolerate a wide range of salinities and temperatures. It has long been used as a model system to study embryonic development because its embryo has advantages for studying both asymmetric cell division and classical spiral development. Details of the developmental stages of *I. obsolete*, such as cleavage stage, post-cleavage stage larvae, and fully developed veliger, can be found in the articles [211].

Notch signaling components have been identified in Ilyanassa, including *IoDelta*, *IoNotch,* and *IoSuH*. Their transcripts are widely expressed at the early cleavage stage, but differ at the subcellular level [79]. For example, within the first 10 h of development, *IoNotch* is ubiquitously expressed, *IoDelta* is strongly expressed in the 4d daughters 4dL and 4dR as well as the 3c and 3d derivatives, whereas *IoSuH* is abundant near the nucleus of the 4D cell and in the bilaterally paired teloblast cells 4dL1M and 4dR1M, with an interesting enhanced perinuclear expression in the macromeres 2C and 2D during the 2q stage. Veligers treated with DAPT show a range of defects that include abnormally formed endoderms and structures that depend on 3D/4d induction-reduced velum, reduced foot, and small or no shell formation. Veligers resulting from the injection of *IoDelta* siRNA generally mimic the defects observed in DAPT-treated larvae. Based on the above data, it was shown that the inhibition of Notch signaling affects endoderm formation and many of the cell fates induced by 3D/4d, as well as the 4d mesendodermal lineage. *I. obsolete* is also an important model for studying comparative neurobiology and the events of eye regeneration [80]. However, the specific functions of the Notch signaling pathway in regulating neurogenesis are still under investigation.

#### 3.7.2. Bivalvia (Pelecypoda): *Crassostrea gigas* (*C. gigas*)

Oyster is a collective name for many bivalve mollusks. The Notch signaling pathway and related miRNAs are closely associated with transplantation immunity, shell coloration, and gonadal differentiation in many common oyster species [e.g., *C. gigas*, *Pinctada fucata martensii* (*P. f. martensii*), *Crassostrea hongkongensis* (*C. hongkongensis*)] [212,213]. The *C. gigas species* has an open circulatory system that continuously and directly interacts with the external environment [81]. It has been proposed that the white shell variant of *C. gigas* may use ‘endocytosis’ to downregulate Notch levels to prevent shell pigmentation, as Notch-related genes bind calcium ions as an upstream component of the shell coloration decision process [82]. Oyster hemocytes, thought to be the vertebrate counterpart of leukocytes, are involved in humoral and cellular immune responses, including antimicrobial peptide synthesis, encapsulation, and phagocytosis [83]. Notch components *CgNotch*, *CgDelta*/*CgJagged*, and *CgHes1* have been found in *C. gigas* [81]. Both *CgNotch* and *CgHes1* increased significantly after *CgIkaros-like* was silenced by siRNAs. Thus, *CgIkaros-like* may bind to the Notch signaling pathway to regulate hematopoiesis in *C. gigas* [83]. However, whether there are other conserved Notch signaling components that are expressed or function as neurodeterminants in *C. gigas* requires further investigation. Notch-related components are involved in mollusk shell pigmentation and color patterning. Reported in *Meretrix meretrix* (*M. meretrix*) and *C. gigas* among the Notch signals components in *M. meretrix* are Delta and Notch. The Notch pathway is involved in shell pigmentation in a gene-dose-dependent pattern. That is, the darker the clam shell color, the higher the Notch expression in its mantle. There were significant differences in shell color patterns in *M. meretrix* in addition to shell pigmentation, so the researchers hypothesized that the Notch signaling pathway may be involved in the formation of shell color patterns [84]. In *P. f. martensii Pm-novel-miR-63*, a species-specific miRNA, regulates transplantation immune through the Notch signaling pathway. Notch signaling components found in *P. f. martensii* include NOTCH1-3, DTX, and TBCE [85]. There are studies that white-shell oysters could employ endocytosis to downregulate Notch levels and lead to melanoblasts apoptosis, preventing pigmentation [82]. In addition to this, Notch-related genes (Delta, Jagged-2, E3 ubiquitin-protein ligase) are upregulated in the albino juvenile phenotype in *Pinctada margaritifera* (*P. margaritifera*) [86].

#### 3.7.3. Cephalopoda

Coleoid cephalopods, including squid, cuttlefish, and octopus, have large and complex nervous systems. They are the only branch outside of vertebrates to have evolved large brains and camera-type eyes. Their brains are primarily effective in the visual system [214]. Cephalopods have extraordinary regenerative abilities, e.g., *Sepia officinalis* (*S. officinalis*) rebuilds lost arms, the *Octopus vulgaris* (*O. vulgaris*) pallial nerve regenerates and restores function, and in addition to arms, octopuses’ nerves can regenerate corneal tissue [215,216].

In *Doryteuthis pealeii* [*D. pealeii* (often called the Woods Hole squid)], Notch maintains a progenitor pool in the cephalopod retina. It has been shown that Notch signaling is required for *Doryteuthis pealeii* (*D. pealeii*) to maintain retinal progenitor cell identity. The Notch signaling components identified in *D. pealeii* are Delta/Jagged, *DpNotch,* and *DpHes-1*. Notch is expressed in the ventral side of the placode and the surrounding extraocular tissue, and Hes is expressed in the placode. All Delta and Hes members except *Isogroup00902* mirror Notch expression. DAPT-treated *D. pealeii* embryos show microphthalmic and a lack of retinal pigmentation. In sectioned samples, the retina was completely disorganized and no photoreceptor cells could be detected [87]. Loss of the retinal progenitor cell marker *DpSoxB1* from the retina in DAPT-treated Doryteuthis pealeii embryos, expansion of the expression of the neuroblast marker *DpEphR,* and loss of *DpNotch* and Hes expression have also been reported. The above data suggest that Notch signaling can regulate the retinal cell cycle and cell fate [88].

### 3.8. Arthropoda

The largest phylum Arthropoda consists of more than one million known invertebrate species in four subphyla: Uniramia, Chelicerata, Crustacea (crustaceans), and Trilobita (trilobites). Uniramia, the largest arthropod subphyla, comprises five classes, including Insecta [(insects, such as the commonly used laboratory model *Drosophila melanogaster* (*D. melanogaster*) and *Tribolium castaneum* (*T. castaneum*)] and Myriapods (which include centipedes and millipedes). Chelicerata contains three classes, including arachnids and horseshoe crabs. Notice that the relationships of major arthropod clades have long been contentious, with classifications of Arthropoda still being updated and amended [217].

In arthropods, neurogenesis takes place within a broad ventral domain called the ventral neuroectoderm (VNE), which is competent to form both ectoderm and neural precursor cells. There is great variation in the way neural precursor cells are formed in arthropods. In the insect VNE, Delta–Notch signaling mediates the formation of neuroblasts (NBs) and epidermal cells [218]. Many organisms can regenerate, and arthropod limbs are no exception, and the Notch pathway plays diverse and fundamental roles in this process [219]. In addition to the functions mentioned above, Notch signaling controls body segmentation in arthropods [220]. Thus, this section will briefly and primarily focus on the involvement of Notch signaling in arthropod neurogenesis, regeneration, and segmentation using a few species.

#### 3.8.1. Uniramia: *D. melanogaster*, *T. castaneum,* and *S. maritima*

Notch signaling was first described in 1914 when John S. Dexter discovered notches in the wings of flies, and the lack of redundancy in the components of the Notch pathway in *Drosophila* makes it a valuable model for developmental neurobiology and other fields [221,222,223]. In *Drosophila*, two Notch ligands have been identified, Dl and Ser, both of which are type I transmembrane structural domain proteins containing multiple EGF repeats in the extracellular structural domain [89,90,91]. A wide variety of tissues express Serrate mRNA, and its expression pattern is tightly regulated both temporally and spatially, mainly in the ventral nerve cord and within the supra-esophageal ganglia (brain hemispheres). Embryonic lethal Serrate mutations exhibit epidermal and neuronal defects, and the phenotype of the Serrate lethal allele is characterized primarily by the absence of large areas of dorsal or ventral cuticle and by the expression of missing connections or breaks in the longitudinal junctions between segmental ganglia [89]. Delta is one of the neurogenic genes of *D.melanogaste* and it is expressed in the intestine in addition to the neurogenic regions. Delta function is required for the proper separation of neuronal and epidermal cell lineages [94,95,96]. *Drosophila* has only one Notch receptor. Defects in the Notch locus result in abnormal embryonic development, and homozygous Notch-deficient embryos show hypertrophy of the nervous system, notched wings, thickened wing veins, and minor bristle abnormalities [92]. Thus, the most classical function of Notch is to maintain *Drosophila* wing development, and when mutated, Notch affects cell proliferation, vein differentiation, and wing margin formation [93]. In *Drosophila*, the transcription factor Su(H) regulates the expression of Notch target genes. The Su(H) protein was detected in pre-blastoderm embryos and was present throughout embryogenesis [94]. In Su(H) mutant cells, ato (proneural gene atonal) expression was initiated normally but subsequently persisted in too many cells, leading to ectopic R8 (photoreceptor cell) cell differentiation and neural hypertrophy [95]. The E(spl) gene serves as a direct target of Su(H) transcriptional activation. E(spl) is a complex locus that includes seven genes encoding closely related basic bHLH motif transcription factors (m8, m7, m5, m3, mβ, mγ, and mδ) and two genes encoding non-bHLH proteins [95]. E(spl) is expressed in the embryonic/larval/pupal eye disk/gut/ovary/pupal CNS/pupal leg disk/larval trachea/pupal wing disk during Drosophila development. Spontaneous dominant mutations of E(spl) in *Drosophila* enhance the small eye phenotype caused by Split (N^spl^, a recessive allele of Notch) and the proliferation of the embryonic nervous system at the expense of the larval epidermis. Furthermore, loss of E(spl) bHLH function produces a neurogenic phenotype typical of E(spl)-C defects. Thus, the classical Notch pathway also plays an important role in *Drosophila* neurogenesis.

Four components of γ-secretase have been demonstrated in *Drosophila*: Ps, Nct, Aph-1, and Pen-2 [96]. Ps and Aph-1 have only one homolog in *Drosophila*. Ps are a highly conserved family of proteins. The *Drosophila* presenilin protein (Dps) is widely expressed during development, with the highest levels in neurons within the larval CNS. Loss-of-function mutations in Dps cause early pupal lethality with underdeveloped eye and wing imaginal discs and defects in neuronal differentiation [97]. Meanwhile, flies lacking Aph-1 function exhibit a phenotype similar to that in the loss of Notch signaling: extensive neural proliferation, notched wings, and thickened wing vein phenotypes. This function of Aph-1 is independent of its role in regulating γ-secretase activity, but may involve downregulation of the activity of the uncleaved Psn holoprotein [98]. Mutant clones of Nct also produce typical Notch phenotypes, such as large notches in the wing margin, thickening of the wing veins, and the loss or duplication of sensory bristles. In addition, embryos lacking maternal and zygotic Nct activity show only a small patch of cuticle formation on the dorsal side, and Nct-mutant follicle cell clones show an identical phenotype to clones of null mutations in Notch or Psn, causing an increase in cell number, a decrease in cell size in mutant clones, expressing high levels of the immature follicle cell marker Fasiclin III (Fas III), and never expressing any markers of differentiated follicle cell types [99]. Taken together, the Notch pathway and its regulation by the components of γ-secretase are required for neuronal differentiation and affect the ability of subsequent signaling.

Neurogenesis has been described in several arthropods besides *Drosophila*, and *T. castaneum* has two E(spl) homologues, E(spl)1 and E(spl)3. Tc E(spl)1/3 are expressed in the embryonic cephalic and trunk neuroectoderm, and their expression depends on both Notch and Ash (achaete–scute homolog). Knockdown of Notch causes severe defects in Tribolium embryogenesis, and the E(spl) RNAi phenotype resembles the neurogenic Notch lof phenotype. In addition, TcE(spl)1/3 have the ability to generate gain-of-function phenotypes identical to those of *Drosophila* E(spl) genes when overexpressed in fly tissues [100]. Other Notch signaling components (e.g., Delta and Notch) in *T. castaneum* are also involved in oogenesis [101].

The Notch signaling pathway involved in neural precursor formation is conserved in arthropods despite differences in arthropod neural precursor formation. In the centipede *S. maritima* (Myriapoda; Chilopoda), the Notch ligand *StmDelta* showed a dynamic expression pattern in the ventral neural ectoderm throughout neurogenesis, and the *StmNotch* is commonly expressed throughout neural development. It is especially transiently expressed at high levels in the lateral region of the ventral neural ectoderm facing the limb buds, and this situation is similar to the spider *CsNotch*. These expression positions suggest that they may play a role in neural precursor specification [103]. In addition, *S. maritima* is unique among the arthropods in showing many cycles of expression of Notch and other segmentation genes within the “presegmental ectoderm” so far. And all these Notch signaling components are all expressed in the posterior disc of *S. maritima* embryos, in radial patterns centered on the proctodeum. This reflects the fact that *StmNotch* and *StmDelta* play a role in the early stages of segmentation. Notch-mediated signaling is required for segment pattern formation in other arthropods, suggesting that the ancestral arthropod segmentation cascade may have involved a segmentation oscillator that utilized Notch signaling [104].

There are relatively few studies on the involvement of Notch signaling in arthropod regeneration, with the following being the main examples. The *Drosophila* midgut epithelium undergoes continuous regeneration that is sustained by multipotent intestinal stem cells (ISCs) underneath. Notch signaling has dual functions to control ISC behavior: it slows down the ISC proliferation and drives the activated ISCs into different differentiation pathways in a dose-dependent manner. Thus, Notch signaling is indirectly involved in the regeneration of the *Drosophila* intestine [102]. Damage to the central nervous system in *Drosophila* induces glial cell proliferation, a result realized by pros-Notch [224]. In addition to this, the involvement of Notch signaling in regeneration also occurs in other arthropods such as *Harmonia axyridis* (*H. axyridis*) in leg regeneration[225].

#### 3.8.2. Chelicerata: *Cupiennius salei* (*C. salei*)

Analysis of *C. salei* neurogenesis suggests that groups of cells rather than single cells are recruited for neural fate. In *C. salei*, segments are sequentially generated from a posterior growth zone. Delta–Notch signaling is involved in the development of ventral neural ectoderm and body segment formation in *C. salei.* Delta–Notch signaling mediates lateral inhibition in the ventral neuroectoderm of the spider in the same manner as in *Drosophila*. The Notch pathway components in *C. salei* include *CsDelta1*/*CsDelta2*/*CsNotch*/*CsSu(H)-1*/*Cs-Su(H)-2*/*CsHairy*/*CsPsn*. *CsDelta1*, *CsDelta2* and *CsNotch*, and they are expressed in a spatiotemporal pattern during ventral nerve ectodermal neuronal cell formation, and they are also expressed in the fragment. Phenotypic analysis of double-stranded RNA by injection of *CsDelta1*, *CsDelta2* and *CsNotch* has shown that the invagination sites are missing to different degrees in embryos, and the neuroectoderm forms bulges because the cells that normally invaginate occupy a space in the apical layer. A strong upregulation of *CsASH1* expression can be observed. In addition to this, serious defects arise in the segmentation pattern and the formed segmentation boundaries. *CsSu(H)-1*/*CsSu(H)-2*/*CsPsn* expression in the prosomal and opisthosomal segments and in the growth zone has been noted. Su(H) RNAi and Psn RNAi have similar phenotypes in *CsHairy* expression: they are neither in the nervous system nor in the growth zone hairy expression [105,106].

#### 3.8.3. Crustacea: *Penaeus vannamei* (*P. vannamei*) and *Litopenaeus vannamei* (*L. vannamei*)

The pacific white shrimp, *P. vannamei*, is one of the most commonly cultured shrimp species worldwide. The most well-established functions of the Notch signaling pathway include the regulation of neurogenesis, cell fate, and angiogenesis, but a growing body of recent research suggests that this pathway is also involved in regulating immune defense. The Notch signaling pathway is thought to be involved in the immune defense of *P. vannamei* and *L. vannamei*, but the relationship between Notch components and this immune defense needs to be further explored. Notch signaling homologs have been identified in *P. vannamei*, and the Notch signaling homologs that have been identified include *PvDelta*, *PvNotch*, *PvCsl*, and *PvHey2*. *PvDelta* was ubiquitously expressed in the *P. vannamei* tissues, with the highest expression seen in the muscle and lowest in hemocytes. The *Pvdelta* expression profile altered after Vibrio parahaemolyticus, white spot syndrome virus (WSSV), and Lipopolysaccharide (LPS) attack. This result shows that *PvDelta* responds to various pathogen challenges, indicating its role in the shrimp immune response. Moreover, after *PvDelta* knockdown followed by LPS stimulation, the expression of Notch signaling pathway genes (*PvNotch*, *PvCsl*, and *PvHey*) was downregulated and shrimp survival was reduced. These data suggest that *PvDelta* positively regulates the *P. vannamei* Notch signaling pathway in *P. vannamei*. In addition, *PvDelta* improves shrimp resistance to pathogen infection. These findings will provide additional insights into Notch signaling in arthropod immune defenses [107]. In vivo knockdown of *PvCsl* significantly attenuated the expression of the *P. vannamei* hemocyanin small subunit gene (*PvHMCs*), and the transcripts of *PvCsl* and *Pv HMCs* were positively correlated under the invasion of pathogens. This result suggests that *PvCsl* is a key factor in the regulation of *PvHMC* transcription [108].

Notch components identified in *L. vannamei* include *LvNotch*, *LvCsl*, *LvPse2*, *LvAph-1*, *LvPsen1*, *LvCtBP,* and *LvTACE*. The knockdown of *LvNotch* resulted in the upregulation of immune-related protein COP9 signalosome complex subunit 1 (*LvCSN1*) and NF-κB pathway-related gene expression in hematopoietic cells, whereas NF-κB pathway-related gene expression was reduced after *LvCSN1* depletion. It was further shown that shrimp Notch (*LvNotch*) has a negative regulatory effect on the NF-κB pathway by regulating *LvCSN1*, providing new insights into the role of Notch in shrimp immune responses [109].

*LvCsl* was widely expressed in *P. vannamei*, with the highest expression seen in blood cells and lower levels of *LvCsl* expression in muscle, gills, and hepatopancreas. The role of *LvCsl* in shrimp immunity was firstly demonstrated by inducing its expression after pathogen attack, and secondly the knockdown of *LvCsl* reduced some immune-related genes and regulated hemocyte proliferation [110].

### 3.9. Echinodermata

Echinoderms are radially symmetrical coelomate marine invertebrates with a calcareous endoskeleton and a water vascular system which helps operate their small podia. Examples of echinoderms include starfish, brittle stars, sea cucumbers, sea urchins, and sea lilies, which do not have a centralized brain but instead have diffuse neural networks called neuronal networks [226]. The nervous system of echinoderms, with the exception of the Crinoidea class (sea lilies), contains the external and hyponeural systems [227]. The external nervous system, which includes the central nerve ring and the outer radial nerves, has motor and sensory functions, whereas the hyponeural subsystem is the inner layer of the radial nerve cord and is involved in locomotor motor functions [228]. Echinoderms have an amazing ability to regenerate at all stages of life. Among the echinoderms, crinoids (feather stars and sea lilies) are known to possess a high potential for regeneration and are able to regenerate most of their organs [229]. Some species can regenerate their entire body while others regenerate locally [230]. There is evidence that the Notch signaling pathway is involved in the above biological features and mediates embryonic development, as shown in the following examples.

#### 3.9.1. Echinoidea/Sea Urchin

Sea urchins can be good model organisms for studying embryogenesis and neurodevelopment [231]. Notch signaling has been documented in the following sea urchins, including *Hemicentrotus pulcherrimus* (*H. pulcherrimus*), *Paracentrotus lividus* (*P. lividus*), *Strongylocentrotus purpuratus* (*S. purpuratus*), and *Lumbriculus variegatus* (*L. variegatus*). There is evidence that Notch signaling is relatively conserved and is involved in embryonic development, the separation of the non-osteogenic mesoderm (NSM), and early endoderm, neurogenesis, and regeneration in sea urchins.

The morphogenesis of sea urchins follows a broad molecular specification, and it is proposed that the topology of gene regulatory networks (GRNs) control phenotypic evolution, as GRN changes leading to the switching of two stable modes of *Delta–Notch* signaling may be a common evolutionary mechanism for changes in embryogenesis [232]. In addition, PSEN is required for embryonic development in *P. lividus* [233].

Notch signaling members in *H. pulcherrimus* include *HpDelta*, *HpNotch*, *HpSu(H)*, Numb, and Fringe (a modifier molecule in the Notch signaling pathway). *HpNotch* is initially expressed in most of the cells, including veg2 blastomeres, and *HpDelta* is expressed in micromere descendants, while *HpSu(H)* is ubiquitously expressed up to the unhatched blastula stage, with its expression being detected exclusively in the vegetal plate region from the hatched blastula stage and then in the archenteron at the gastrula stage. *HpSu(H)* perturbation results in the disappearance of secondary mesenchyme cells at the tip of the archenteron in the gastrula and pigment cells in the pluteus larva, leading to a defect or atrophy of the foregut in the archenteron at the pluteus stage. This confirms that Notch signaling is required for *NSM* specification and foregut development [111]. DAPT treatment further demonstrated that Notch signaling is involved in the specification process of various types of secondary mesenchymal cells (SMCs). In *S. Purpuratus,* components of the Notch signaling pathway identified in transcriptome analyses include two ligands *SpDelta16128* and *SpSerrate22053*, a Notch protein *SpNotch14131*, two Notch-like receptors *SpNotchlike27006* and *SpNotchlike19810*, *SpHes*, and γ-secretase components *SpPsen* (only one copy is found in sea urchins), *SpAph-1*, *SpPen-2*, and *SpNicastrin19033* [113]. Delta–Notch signaling is also involved in the activation of NSM specification, pigment cell formation, and phagocytosis in *S. Purpuratus* [114,115] and the development of mesoderm and endoderm in *L. variegatus* embryos, as knockdown of either *LvNotch* or *LvFringe* both lead to a reduction in secondary SMCs and a delay in archenteron invagination, with some endodermal gene expression being compromised [119].

Delta–Notch plays a role in neurogenesis in sea urchin embryos, as Delta is specifically expressed in serotonergic neural precursors in sea urchin embryos and is co-expressed with the zinc finger homeobox (zfhx1/z81), which is one of the targets of anti-neural signaling inhibition and which plays a role in the specification of individual anterior neural precursors and in the differentiation of serotonergic neurons [112]. The inhibition of Notch signaling by inhibition of *γ-secretase*, injection of *SpDelta* morpholinos, *CRISPR/Cas9*-induced mutation of *SpDelta, or* miR-124 regulation all result in more neural precursors and neurons [116,117] In fact, Delta–Notch signaling is used by all three neural subtypes as a lineage-restriction mechanism in *L. variegatus* [120]. Thus, Notch signaling limits the number of recruited neural progenitors and regulates the fate of asymmetrically dividing progeny [116]. PSEN is also expressed in neurogenic regions of the sea urchin embryo, such as the oral ganglia innervate and the lower lips of the mouth [118]. PSEN expressed around the gut may be related to neural control, which acts on the ciliary beating of ciliated cells. The co-labeling of PSEN and anti-acetylated tubulin antibody was detected in the ciliary band, which is linked to the gut by sensory and motor neurons in sea urchins [234].

Notch signaling is less well studied for sea urchin regeneration; however, the chemical inhibition of Notch signaling by DAPT leads to inhibition of the regeneration of amputated tube feet and sea urchin spines and exhibits stoichiometry-dependence, suggesting that Notch is essential for these different regenerative processes as well [121].

#### 3.9.2. Asteroidea/Starfish: *Patiria miniata* (*P. miniata*)

Sea stars, or starfish, use their many podia to crawl slowly across most surfaces, making them among the most ambulatory or mobile of all echinoderms. *P. miniata* have a complex internal anatomy containing a calcareous endoskeleton, and in association with the cell there is a system of muscles and ligaments, two systems of coelomic canals, and a complex nervous system including a radial nerve and numerous peripheral nerves [235]. Although sea stars and sea urchins evolved from a last common ancestor, the special mesodermal specification circuit of sea urchins does not exist in sea stars. This mesodermal specification is initiated by the Delta signal emitted by skeletogenic cells; Delta activates the Notch receptor on the surface of future mesodermal cells, thereby controlling mesodermal GCM transcription, some of which is directly required for the subsequent appearance of mesodermal pigment cells [122]. Furthermore, the Delta–Notch regulation of Hex is required to maintain endoderm specification, and specification and maintenance of tube cell fate requires Delta–Notch signaling [123,124].

#### 3.9.3. Ophiuroidea/Brittle Stars

Brittle stars, the seafloor ecosystem engineers, are closely related to starfish, but they are a completely different species. The brittle star *Ophioderma brevispina* (*O. brevispina*) has become an important model for regeneration studies in echinoderm, and the Notch pathway is required for proper arm regeneration in *O. brevispina.* Major components of the Notch pathway in *O. brevispina* transcriptome data include Delta/Serrate, ADAM10/17, Notch, RBPJ, Nct, Psen1, two Notch target genes of the Hes family, Notch signaling modulator Deltex, and Notchless [125]. Although the specific localization and function of each signaling molecule in starfish is still not well understood, the inhibition of DAPT resulted in a significant reduction in the total length of arm growth and a reduction in the number of segments in newborn arms, but they still contained all major radial organs [126]. Crosstalk between the Notch pathway and other signaling pathways also occurs during regeneration.

#### 3.9.4. Holothuroidea/Sea Cucumbers

In *Apostichopus japonicus* (*A. japonicus*), members of the Notch signaling pathway are expressed in melanocytes and appear to be upregulated in melanoma cell lines. Higher levels of DEGs were present during the pigmentation of sea cucumber macros. The involvement of Notch signaling in the pigmentation process has previously been reported in a gene-dose-dependent manner in clams, but not in sea cucumbers [84]. The researchers hypothesized that the Notch signaling pathway may be an upstream component of the pigmentation process in sea cucumbers, determining the location and boundaries of pigmentation [127]. Sea cucumbers have an amazing ability to regenerate, and they are able to regenerate new internal organs after most of them have been emptied [236]. Notch signaling molecules currently measured in *A. japonicus* include Serrate/Delta, Deltex, Fringe, Notch, MAML, Psen, Csl, and CtBP. However, the involvement of the Notch signaling pathway in sea cucumber regeneration has not been specifically addressed [128].

### 3.10. Chordata

The phylum Chordata includes all vertebrates and many invertebrates, and these chordates can be divided into three subphyla animals with different general characteristics: Cepahlochordata (cephalochordates), Urochordata (also known as the subphylum Tunicata, tunicates), and Vertebrata (vertebrates). But at some point in a chordate’s life, they all have a stiff dorsal support called the notochord, which is flanked dorsally by the nerve cord. Here, we will only give a brief introduction to three species of ascidians [237].

Ascidians or sea squirts are marine invertebrates belonging to the subphylum urochordate/tunicate, including solitary and colonial species. Colonial ascidians are good models for studying the evolution of coloniality, self–non-self incompatibility systems, and whole-body regeneration [238]. In colonial ascidians, such as *Botrylloides leachii* (*B. leachii*), the entire body can regenerate from small fragments of extracorporeal vasculature, and these animals are the only chordates that are capable of whole-body regeneration [239]. Regeneration is probably more limited in solitary ascidians, such as *Ciona intestinalis* (*C. intestinalis*), in which distal body parts can be replaced by proximal parts, as long as the latter contain part of the branchial sac [240]. In *Ciona*, the oral siphon (OS, a muscular tube leading to the mucus-forming pharynx) and the neural complex (the brain and the associated neural gland) are able to regenerate with complete fidelity within about a month after their removal [241]. In short, the range of regenerative abilities within chordates is very broad, from wound healing associated with scarring (humans, mice), to the ability to completely regenerate limbs and the spinal cord without scarring (amphibians), to the regeneration of the entire central nervous system (*Ciona*), to the extreme case of whole-body regeneration (*Botrylloides*). The Notch signaling pathway is involved in the systemic regeneration of *B. leachii leachi* and the regeneration of *C. intestinalis* [129]. In *Ciona*, members of the Notch signaling pathway, including those encoding the Delta and Jagged ligands, two fringe modulators and, to a lesser extent, the Notch receptor, are upregulated during OS regeneration, and regeneration of the OS and other distal structures occurs by epimorphosis involving the formation of a blastema of proliferating cells. Since Notch signaling is involved in maintaining proliferative activity in both the Ciona and vertebrate regenerative blastema, the results suggest a conserved evolutionary role for this pathway in chordate regeneration [130]. In addition, Jerry S. Chen et al. discovered that feedback interaction between miR-124 and Notch signaling regulates the epidermal-peripheral nervous system fate choice in *Ciona* tail midline cells, and that this interaction may be unique to sea squirts [131]. Ectopic expression of miR-124 is sufficient to convert epidermal midline cells into PNS neurons, consistent with a role in modulating Notch signaling as five Notch pathway genes (Notch, Neuralized, and the three *Ciona* Hes genes) are specific targets of *Ciona* miR-124. Furthermore, *CiSu(H)* is also involved in neurogenesis, as blocking Notch signaling in the epidermis by expressing a dominant-negative form of Suppressor of Hairless resulted in the formation of extra ciliated epidermal sensory neurons (ESNs) along the tail midline, and a similar phenotype is also observed when ectopic epidermal expression of miR-124 is induced [131].

Notch signaling components, including *Hrdelta* and *HrNotch*, have been identified in another ascidian, *Halocynthia roretzi* (*H. roretzi*). *HrDelta* is expressed in the precursors of peripheral neurons and adhesive organs (palps), while at the neurula stage, *HrDelta* is widely expressed in the putative neurogenic region. The positive feedback mechanism of *HrDelta* expression operates the fate determination of palps and peripheral neurons. Predominant expression of *HrNotch* in epidermal and neural cells is a common feature of chordate Notch genes. At later stages of development, *HrNotch* mRNA was more evident in the central nervous system [132]. Overexpression of *HrNotch* by injecting the synthesized mRNA into fertilized eggs resulted in defects in neural tube formation and suppression of the formation of brain vesicles, palps, and the epidermal PNS. These data suggest that Notch signaling components in the ascidian play a crucial role in the development of the nervous system and that it affects the fate choice between palps and epidermis and between peripheral neurons and epidermis within the neurogenic regions of the surface ectoderm [133].

## 4. Conclusions

**Figure 2:**
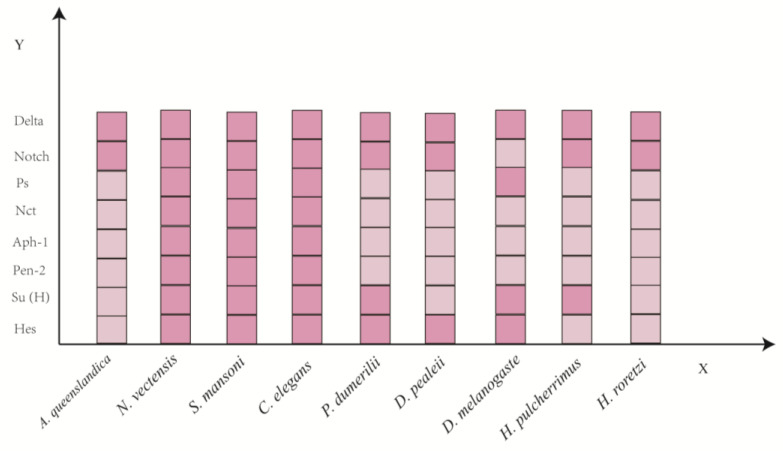
Expression of core components of the Notch signaling pathway in invertebrates. The expression of core components of the Notch signaling pathway varies in lower organisms. Pink rectangles represent the corresponding genes are expressed in the species, while light-pink rectangles indicate that the corresponding genes are not examined/expressed in the species based on current studies. In the axes, the X-axis represents the rank of the species from left to right from low to high, and the Y-axis represents the core components of the Notch signaling pathway. Delta, ligand; Notch, receptor; Ps, presenilin; Nct, nicastrin; Aph-1, anterior pharynx-defective 1; Pen-2, presenilin enhancer 2; Su(H), Suppressor of Hairless; Hes, Hairy and enhancer of Split; *A. queenslandica*, *Amphimdeon queenslandica*; *N. vectensis*, *Nematostella vectensis*; *S. mansoni*, *Schistosoma mansoni*; *C. elegans*, *Caenorhabdits elegans*, *P. dumerilii*, *Platynereis dumerilii*; *D. pealeii*, *Doryteuthis pealeii*; *D. melanogaster*, *Drosophila melanogaster*; *H. pulcherrimus*, *Hemicentrotus pulcherrimus*; *H. roretzi*, *Halocynthia roretzi*.

The Notch signaling pathway is a highly evolutionarily conserved cell signaling system in metazoans, and most Notch pathway components were already present in the Urmetazoa (the last common ancestor of all metazoans), although some were repeatedly lost in various bilaterian species, such as Mastermind (Figure 2). In fact, Notch pathway components were also present in a more primitive protozoan, the choanoflagellates [10].

The Notch pathway plays important roles in embryonic development, neurogenesis, segmentation, immune response, intestinal homeostasis, and tissue regeneration through regulating stem cell maintenance, cell fate choices, cell apoptosis, cell–cell communication, and so on [44,52,242,243,244]. At first glance, the extreme versatility and pleiotropy of Notch signaling seems to make the task of reconstructing an evolutionary history of its involvement in metazoan development rather daunting. Although many reviews have summarized the function of Notch from different perspectives, there are still many areas where the function of Notch components is not well understood [3,10]. This review summarizes the past and present studies that have revealed some of the mechanisms underlying Notch signaling in invertebrate neurodevelopment and regeneration by using a few relatively typical species [44,52,245]. This is valuable for the comparative analysis of Notch function in the same organ/tissue and in the same processes of different species, as it allows the specific advantages of each model system to be exploited to highlight both conserved and divergent functions across species barriers and is also capable of discovering unexplored areas, such as how the Notch signaling pathway acts during regeneration and is independently co-opted for regeneration [51,130]. Future studies are expected to clarify these issues by using different regenerative animal models and comparing analyses.

## Figures and Tables

**Table 1:** Expression and function of Notch components in invertebrates.

Species Name	Expression	Function	Ref.
**Demospongea (*A. queenslandica*** **)**			
*AmDelta1-5*	Embryos and larvae	Embryonic development and neurodevelopment	[40,41,42]
*AmNotch*	Globular cells, anterior polar cells, ciliated cells		
*AmHLH1*			
**Hexactinellida (*A. vastus*)**			
*AvADAM10/17* *AvFurinL* *AvDelta* *AvNotch* *AvSuH* *AvPEN2* *AvAPH1* *AvPSEN2* *AvNCSTN* *AvNCoRL*			[40,41]
**Hexactinellida (*O. minuta*)**			
*OmADAM10/17* *OmFurinL* *OmDelta* *OmNotch* *OmSuH* *OmPEN2* *OmAPH1* *OmPSEN2* *OmNCSTN* *OmNCoRL*			[40,41]
**Homoscleromorpha (*O. carmela*)**			
*HsADAM10/15/17* *HsFurin* *HsJag1/Jag2* *HsDDL1/DDL3/DDL4* *HsNotch1* *HsSuH*			[40,41]
**Cnidaria**			
**Hydrozoa (*H. vulgaris*)**			
*HyJagged*	Boundary between bud and parent	Regeneration and development	[43,44,45]
*HvNotch*	Ectodermal and the endodermal		
*HvSu(H)*			
*HyHes-1*	Regenerating heads and tentacle buds		
*Hvpen-2*			
**Anthozoa (*N. vectensis*)**			
*NvDelta/NvJagged*	Oral ectoderm and endoderm of the planula stage	Neurodevelopment and regeneration	[46,47,48]
*NvNotch*	Neural structure, pharyngeal endoderm, gastrula and early planula		
*NvSuH*	Endodermal plate and planula		
*NvHes1*	Ectoderm of the gastrula stage		
*NvHes2*	Oral ectodermal domain		
*NvHes3*	Aboral ectoderm of the gastrula and early planula		
*NvHes4*			
**Anthozoa (*A. cervicornis and A. palmata*)**			
Delta/Delta-like/Jagged Notch Hairy/E(Spl)/Su(H) E3 ubiquitin ligase MIB Numb		Growth	[49]
**Anthozoa** **(*O. faveolata*)**			
Delta/Jagged ADA NOTCH DTX PEN NICA PSN APH EP300 RBPJL HAIRLESS HES		Innate immune defense	[50]
**Platyhelminthes**			
**Turbellaria (*D. japonica*)**			
*DjNotch1–6*	Parenchymal tissues and blastema site	Neurodevelopment and regeneration, eye point development	[51]
*DjRbpj*			
*DjHes*			
**Turbellaria (*S. mediterranea*)**			
*SmedNotch1, SmedNotch2, SmedNotch4*	All notch components are expressed in discrete neural populations throughout the brain and in regenerating tissues.	Parenchymal tissues and the blastema site, eye point development.	[52,53]
Su(H)			
Atoh			
Coe			
Fer3l-1			
Hes (Hesl-3)			
Sim			
**Trematoda (*S. mansoni*)**			
*SmJagged/SmSerrate * *SmNotch* *SmSu(H) * *SmHes* *SmSmart* *SmGroucho* *SmSkip* *smPresenilin* *SmNicastrin* *SmAph-1 * *SmPen-2* *SmFurin* *SmAdam 17/SmKuzbanian* Notchless Numb Dishevelled WWP1	All identified Notch signaling components are present in all life cycles of the S. mansoni	Oogenesis and embryogenesis	[54]
**Cestoda (*E. granulosus*)**			
Delta Jagged Notch Presenilin1 Aph1 Pen-2 Su(H) Dishevelled Numb Metalloproteinase domain-containing protein E3 ubiquitin-protein ligase RNF167 ADAM 17-like protease E1A/CREB-binding protei SNW domain-containing protein 1 C-terminal-binding protein Groucho Histone deacetylase 1/2 Furin-like protease 1	They are expressed at all developmental stages.	Mitotic cell division and proliferation	[55,56,57,58]
**Nemathelminthes**			
**Nematoda (*C. elegans*)**			
Lag-2(Sel-3)/Apx-1/Arg-1/Dsl-1	IL2 neurons	Neurogenesis, embryonic development, normal spawning and vulva development	[59,60,61,62,63,64,65,66]
Lin-12/Glp-1	Larval RIG neurons, somatic gonadal, and vulvar profiles		
Sel-12/Hop-1	Early blastomeres		
Aph-1/Aph-2			
Pen-2			
Lag-1/Sel-8(Lag-3)			
**Annelida**			
**Earthworms (*P. excavates*)**			
*Pex-DeltaD/Pex-Serrate*		Early bidirectional regeneration, regeneration inducing	[67,68,69]
*Pex-Notch*	Anterior regeneration		
*Pex-Hes1A*			
*Pex-Hes4A*	Regenerated head and tail tissues		
*Pex-Hes1B*	Intact tissues and throughout bidirectional regeneration		
*Pex-Hey*			
**Polychaeta (*P. dumerilii*)**			
*Pdu-Delta^tv1^/Pdu-Delta^tv2^*	Chaetal sacs and brain cells	Formation of chaetal sacs, proneural and neuronal specification, larval development, posterior growth, and caudal regeneration	[70,71,72,73,74]
*Pdu-Jagged*	Mesodermal posterior stem cells		
*Pdu-Notch*	Chaetal sacs and brain cells		
*Pdu-SuH*	Chaetal sacs and brain cells		
*Pdu-Presenilin*			
*Pdu-Hes1-15*	Central and peripheral NS		
*Pdu-Twist*	Posterior growth zone and in mesodermal anlagen and developing muscles		
*Pdu-Nrarp*			
*Pdu-Numb*			
*Pdu-Fringe*			
**Polychaeta (*C. teleta*)**			
*CapI-Delta*	Terminal growth zone, ventral nerve cord, chaetal sacs	Development of hair follicles, neurogenesis, segmentation	[75,76]
*CapI-Notch*	Chaetal sacs, terminal growth zone, and ventral nerve cord		
*CapI-hes1*	Mesoderm		
*CapI-hes2*	Brain, pharynx, ventral nerve cord, foregut, and segmental tissue		
*CapI-hes3*	Brain, pharynx, ventral nerve cord, foregut, and segmental tissue		
*CapI-hesr1*			
*CapI-hesr2*			
**Clitellata (*H. robusta*)**			
*Hro-notch*	Both expressed in various cells of the early embryo	Ganglion morphogenesis, posterior elongation and segmentation	[77,78]
*Hro-hes/Csa-hairy*			
**Mollusca**			
**Gastropoda (*I. obsoleta*)**			
*IoDelta*	Transcripts are widely expressed at the early cleavage stage	embryonic development	[79,80]
*IoNotch*			
*IoSuH*			
**Bivalvia (*C. gigas*)**			
*CgDelta/CgJagged* *CgNotch* *CgHes1*		Haematopoiesis, transplantation immunity	[81,82,83]
**Bivalvia (*M. meretrix*)**			
Delta	Mantle	Shell pigmentation, color patterning	[84]
Notch			
**Bivalvia (*P. f. martensii*)**			
NOTCH1-3 DTX TBCE		Regulates transplantation immune	[85]
**Bivalvia** **(*P. margaritifera*)**			
Delta Jagged-2 E3 ubiquitin-protein ligase		Pigmentation	[86]
**Cephalopoda (*D. pealeii*)**			
Delta/Jagged	Apical side of the retinal epithelium	Essential for maintaining retinal progenitor cell identity	[87,88]
*DpNotch*	Ventral side of the placode and surrounding extraocular tissue		
*DpHes-1*	Entire placode		
**Arthropoda**			
**Uniramia (*D. melanogaste*)**			
Serrate/Delta	Ventral nerve cord and within the supraesophogeal ganglia (brain hemispheres) neurogenic regions and intestine	Wing development, embryonic development, regeneration and neurodevelopment	[89,90,91,92,93,94,95,96,97,98,99]
Notch			
Psen	Neurons		
Nct			
Aph-1			
Pen-2			
Su(H)	Pre-blastoderm embryos		
E(spl)	Embryonic/Larval/pupal eye disk/pupal CNS/Gut/Ovary/pupal leg disk/Larval trachea/pupal wing disk		
**Uniramia (*T. castaneum*)**			
E(spl)1/E(spl)3	Embryonic cephalic and trunk neuroectoderm	Immune defense	[100,101,102]
**Uniramia (*S. maritima*)**			
*StmDelta*	Ventral neural ectoderm and the posterior disc	Neural precursor specification and segmented	[103,104]
*StmNotch*	Lateral region of the ventral neural ectoderm		
*StmHes1-4*	Posterior disc		
**Chelicerata (*C. salei*)**			
*CsDelta1/CsDelta2*	Ventral nerve ectodermal neuronal and fragment	Development of ventral neural ectoderm and body segment formation	[105,106]
*CsNotch*	Ventral nerve ectodermal neuronal and fragment		
*CsPsn*	Prosomal, opisthosomal segments, and growth zone		
*CsSu(H)-1/CsSu(H)-2*	Prosomal and opisthosomal segments and growth zone		
*CsHairy*	Ventral nerve ectodermal neuronal and fragment		
**Crustacea (*P. vannamei*)**			
*PvDelta*	Universal expression	Immune defense	[107,108]
*PvNotch*			
*PvCsl*			
*PvHey2*			
**Crustacea (*L. vannamei*)**			
*LvNotch*		Immune responses and regulates hemocyte proliferation	[109,110]
*LvCSL*	Universal expression		
*LvPsen2*			
*LvAph-1*			
*LvPsen1*			
*LvCtBP*			
*LvTACE*			
**Echinodermata**			
**Echinoidea (*H. pulcherrimus*)**			
*HpDelta*	Micromere descendants	Specification of SMCs, neurogenesis, involved in sea urchin foregut development, non-skeletogenic mesoderm specification	[111,112]
*HpNotch*	Veg2 blastomeres		
*HpSu(H)*	Vegetal plate region, the archenteron		
Numb			
Fringe			
**Echinoidea (*S. purpuratus*)**			
*SpDelta16128/SpSerrate22053*		Embryonic development, neural control Limiting the number of neural progenitor cells and neurons, non-skeletogenic mesoderm (NSM) specification, pigment cell formation, and phagocytosis	[113,114,115,116,117,118]
*SpNotch14131*			
*SpNotchlike27006/SpNotchlike19810*			
*SpPsen*	Uniformly in unfertilized eggs, endodermal and mesodermal regions, around the digestive system, neurogenic regions		
*SpAph-1*			
*SpPen-2*			
*SpNicastrin19033*			
*SpHes*	Mesenchyme blastula stage and the vegetal hemisphere		
**Echinoidea (*L. variegatus*)**			
*LvDelta*	Endoderm, neural progenitor cells of the oral ectoderm, and apical organs	Development of the mesoderm and endoderm, regeneration of tube feet and spines	[119,120,121]
*LvFringe*	Universal expression during germ layer development		
**Asteroidea (*P. miniata*)**			
Delta	Mesodermal progenitors of the central vegetal plate, ectoderm	Development of mesoderm and endoderm	[122,123,124]
**Ophiuroidea (*O. brevispina*)**			
Delta/Serrate ADAM 10/17 Notch RBPJ Nct Psen1 Hes Deltex Notchless		Arm regeneration	[125,126]
**Holothuroidea (*A. japonicus*)**			
Serrate/Delta Deltex Fringe Notch MAML Psen Csl CtBP	All expressed in melanocytes	Participation in the pigmentation process of sea cucumbers	[127,128]
**Chordates**			
**Ascidiacea (*C. intestinalis*)**			
Delta and Jagged Fringe Notch Neuralized *CiSu(H)* Hes		Neurodevelopment, regeneration of the oral siphon (OS)	[129,130,131]
**Ascidiacea (*B. leachii*)**			
Delta Notch Hairy/E(Spl) Blgroucho1 Blgroucho2		Whole-body regeneration	[129]
**Ascidiacea (*H. roretzi*)**			
*HrDelta*	Precursor cells of peripheral neurons and palp	Fate determinations of palps and peripheral neurons	[132,133]
*HrNotch*	Epidermal and neural cells		

## Data Availability

Not applicable.

## Abbreviations

Delta (Dl) and Serrate (Ser), Delta-like ligand (DLL), Jagged (JAG), neurogenic locus Notch homolog protein (Notch), disintegrin and metalloproteinase domain-containing protein (ADAM), Presenilin (Ps), Drosophila presenilin protein (Dps), Nicastrin (Nct), Anterior pharyngeal defect 1(Aph-1) and enhancer 2 (Pen-2), Fasiclin III (Fas III), Suppressor of Hairless [Su(H)], Recombination signal binding protein for immunoglobulin kappa J region (RBPJκ), CBF1/Su(H)/LAG-1 (CSL), Hairy and Enhancer of split (HES), Enhancer of split E(spl), Helix-Loop-Helix (bHLH), Deltex E3 Ubiquitin Ligase 1 (DTX1), E1A/CREB-binding protein (EP300), central nervous system (CNS), ventral nerve cord (VNC), peripheral nervous system (PNS), neuroendocrine–immune (NEI) regulatory network, ventral neuroectoderm (VNE), neuroblast (NB), alternative splicing (AS), extracellular vesicle (EV), whole-mount in situ hybridization (WMISH).
